# Alphavirus replicase and regulatory RNA elements in host interactions and viral vector engineering

**DOI:** 10.1128/jvi.01778-25

**Published:** 2026-06-15

**Authors:** Dan T. Boghici, Danni Yong, Silvia M. Vidal, GuanQun Liu

**Affiliations:** 1 Department of Human Genetics, McGill University 686231, Montreal, Quebec, Canada; 2 The McGill Research Centre on Complex Traits, Montreal, Quebec, Canada; 3 Department of Microbiology and Immunology, McGill University 5620, Montreal, Quebec, Canada; 4 Mark Wainberg Centre for Viral Diseases, McGill University 5620, Montreal, Quebec, Canada; Indiana University Bloomington, Bloomington, Indiana, USA

**Keywords:** alphaviruses, self-amplifying RNA, innate immunity, viral vectors, vaccines, immunotherapy, gene therapy

## Abstract

Alphavirus-derived vector systems have been used for heterologous gene expression for nearly four decades, providing foundational insights into RNA virus replication, host interactions, and genome engineering. The recent success of conventional mRNA vaccines has intensified interest in leveraging the alphavirus replicase and its associated conserved sequence elements (CSEs) to develop self-amplifying mRNA (sa-mRNA) platforms characterized by more sustained antigen expression and dose-sparing. In this minireview, we summarize key advances that define the coordinated functions of the nonstructural proteins and CSEs in viral RNA synthesis and host modulation. We then contextualize the developmental milestones of alphavirus replicons that underpin modern sa-mRNA technologies. We further discuss emerging strategies to engineer replicase functions and RNA architecture in different sa-mRNA applications, while outlining critical gaps in alphavirus biology that currently constrain rational sa-mRNA design. Renewed investigation of nonstructural proteins and CSEs will accelerate optimization of next-generation sa-mRNA platforms and reinvigorate fundamental studies of alphavirus replication, evolution, and host adaptation.

## INTRODUCTION

The *Alphavirus* genus comprises ~32 enveloped, positive-sense RNA viruses with ~11.5–12 kb genomes that are 5′ capped and 3′ polyadenylated (1). These viruses are maintained in enzootic and epizootic transmission cycles between mosquitoes and diverse vertebrate hosts, including non-human primates, birds, and fish (2). Sustained urban transmission requires close human-mosquito cohabitation, sufficient viremia in infected individuals to permit human-to-mosquito transmission, and efficient viral replication in the mosquito midgut following a blood meal (3). Based on disease phenotype and geographic origin, alphaviruses are broadly classified into Old World and New World clades, which predominantly cause arthritogenic and encephalitic disease, respectively (4).

Early foundational studies in alphavirus biology were largely based on the mildly pathogenic Old World viruses, Sindbis virus (SINV) and Semliki Forest virus (SFV). Following several recurrent epidemics in Southeast Asia, Africa, and South America since 2004, chikungunya virus (CHIKV) has emerged as the most clinically relevant alphavirus due to its substantial public health impact (5–8). Acute CHIKV infection typically presents as a febrile illness with rash, arthralgia, and myalgia, with peak viremia occurring 2–3 days post-infection and viral clearance within approximately one week. However, chronic and debilitating musculoskeletal symptoms can persist for months to years (9). Given the nonspecific clinical presentation and co-circulation with other arboviruses, CHIKV infections are likely underreported. In the absence of widespread prophylaxis, epidemic regions are estimated to experience recurrent outbreaks approximately every 6 years, with roughly 8% of the population remaining susceptible (10). A deeper understanding of the alphavirus infection cycle and host interactions is thus needed to define the determinants of viral emergence, disease severity, persistence, and evolving neurotropism (11).

Beyond their clinical relevance, alphaviruses are notable for their remarkable genomic plasticity. Replacement of the structural gene cassette with heterologous sequences enables robust and sustained transgene expression in the absence of infectious particle production. These alphavirus replicon systems form the molecular basis of self-amplifying mRNA (sa-mRNA) vaccines and therapeutics, a platform now largely advanced using backbones derived from the New World virus, Venezuelan equine encephalitis virus (VEEV). However, encephalitic alphaviruses such as VEEV and eastern equine encephalitis virus (EEEV) are emerging threats to human health and have demonstrated potential for aerosol transmission, which can result in more severe disease than mosquito-borne infection (12). Consequently, the risks of laboratory-acquired infection and persisting concerns regarding the potential misuse of New World alphaviruses have led to their classification as biosafety level 3 pathogens.

In this minireview, we summarize the functions of alphavirus nonstructural replicase proteins and conserved regulatory RNA elements that direct viral RNA synthesis and host interactions, and discuss how these components can be engineered for emerging sa-mRNA applications. We also review the historical development of alphavirus-based expression systems and highlight key knowledge gaps in alphavirus biology that currently limit the rational design of improved sa-mRNA backbones.

## ALPHAVIRUS GENOME ORGANIZATION

**Fig 1:**
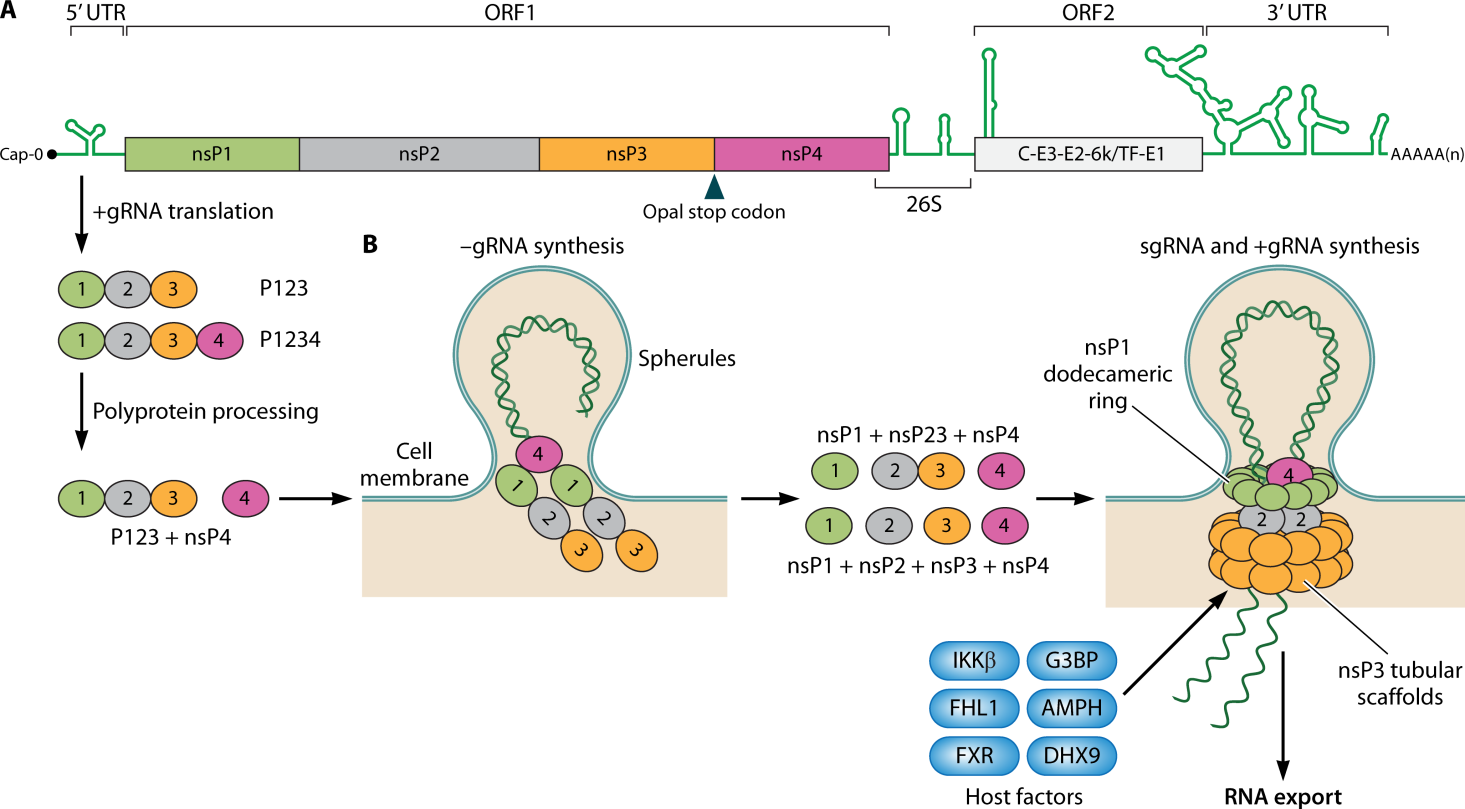
Alphavirus genome organization, replication, and proviral host interactions. (**A**) The positive-sense alphavirus genomic RNA (+gRNA) contains 5′ and 3′ untranslated regions (UTRs), a 3′ poly(A) tail, and two polyprotein open reading frames (ORF1 and ORF2) separated by an intergenic region that overlaps with the 26S subgenomic promoter. Following cap-0-dependent translation of ORF1, the nonstructural polyproteins P123 and, via readthrough at the opal stop codon, P1234 are produced. Proteolytic cleavage by nsP2 at the nsP3|4 junction releases nsP4, which, together with P123, localizes to host membranes and initiates formation of the viral replication organelle known as the spherule. (**B**) At the spherule, the P123–nsP4 complex mediates synthesis of the antigenome (−gRNA). Subsequent processing of P123 into individual nsPs directs production of +gRNA and subgenomic RNA (sgRNA), the latter serving as the template for translation of structural polyproteins (C, E3, E2, 6K/TF, and E1). At the base of the spherule, nsP1 assembles into a dodecameric pore complex that caps and exports newly synthesized RNAs. nsP3 oligomerizes into a scaffold adjacent to the nsP1 pore, contributing to RNA sorting and structural integrity of the replication organelle while serving as a hub for proviral host interactions. Key host factors include stress granule proteins G3BP1/2 (Old World alphaviruses) and FXR1/2 (New World alphaviruses), as well as amphiphysin (AMPH), which promote spherule assembly. The LIM domain protein FHL1 supports replication of CHIKV and ONNV in muscle and joint tissues, while the host RNA helicase DHX9 contributes to viral RNA synthesis. Phosphorylation of nsP3 by IKKβ enhances negative-strand RNA synthesis and virion assembly.

Alphavirus genomes encode two open reading frames that express four nonstructural proteins (nsP1–4) and six structural proteins (C–E3–E2–6k/TF–E1) (13) (Fig. 1A). The 5′ open reading frame (~7 kb) encodes the viral nonstructural polyprotein, which is translated primarily as P123 and through readthrough of an opal stop codon (UGA) at approximately 10%, as P1234 (14). The viral protease nsP2 mediates proteolytic processing of the polyprotein to yield the mature nonstructural proteins nsP1–4, which collectively orchestrate viral RNA replication and transcription while also modulating host responses such as antiviral innate immunity and apoptosis in vertebrate cells (15). The intact P123 precursor is required for the synthesis of full-length negative-strand RNA, which is directed by the virally encoded RNA-dependent RNA polymerase (RdRp), nsP4. Subsequent cleavage events release nsP1–3 and enable transition to positive-strand genomic and subgenomic RNA synthesis (16–18) (Fig. 1B).

An internal 26S promoter on the antigenome directs synthesis of the subgenomic RNA, from which the structural proteins (capsid, E3, E2, 6K/TF, and E1) are translated as two polyproteins. The transframe (TF) protein is generated through −1 ribosomal frameshifting within the 6K coding region, producing an alternative structural polyprotein that terminates upstream of E1. Capsid undergoes rapid autocleavage and remains cytosolic, while E3 directs the remaining polyprotein to the secretory pathway, through which E1 and E2 are glycosylated (19–21). E3 chaperones E2 as the p62 precursor, shielding the E1 fusion loop until furin-mediated cleavage generates mature, fusion-competent glycoprotein spikes (22, 23). In addition, 6K and TF promote efficient budding and virion assembly, and TF has been further implicated in antagonism of the host type I interferon (IFN) response (24–26). Mature virions consist of an enveloped nucleocapsid displaying 240 E1–E2 heterodimers arranged into 80 trimeric spikes that mediate receptor-dependent entry and pH-triggered membrane fusion (27–30).

## ALPHAVIRUS NSP1 AND NSP2 COORDINATE REPLICATION AND IMMUNE EVASION

nsP1 is a multifunctional membrane-associated protein that anchors the viral replication machinery to host membranes. Its amphipathic helix inserts into anionic lipid domains at the plasma membrane to drive the formation of bulb-like evaginations known as spherules (31, 32) (Fig. 1B), with species-specific requirements for cholesterol-rich microdomains that more greatly affect CHIKV than SINV (33). At the spherule neck, nsP1 assembles into a dodecameric pore complex that exports newly synthesized viral RNAs to the cytosol (34). These membrane-bound compartments sequester double-stranded RNA (dsRNA) replication intermediates from innate immune sensors such as RIG-I and MDA5, which nevertheless contribute to IFN induction during alphavirus infection (35–37). However, the precise ligands responsible for activating these sensors remain incompletely defined.

**Fig 2:**
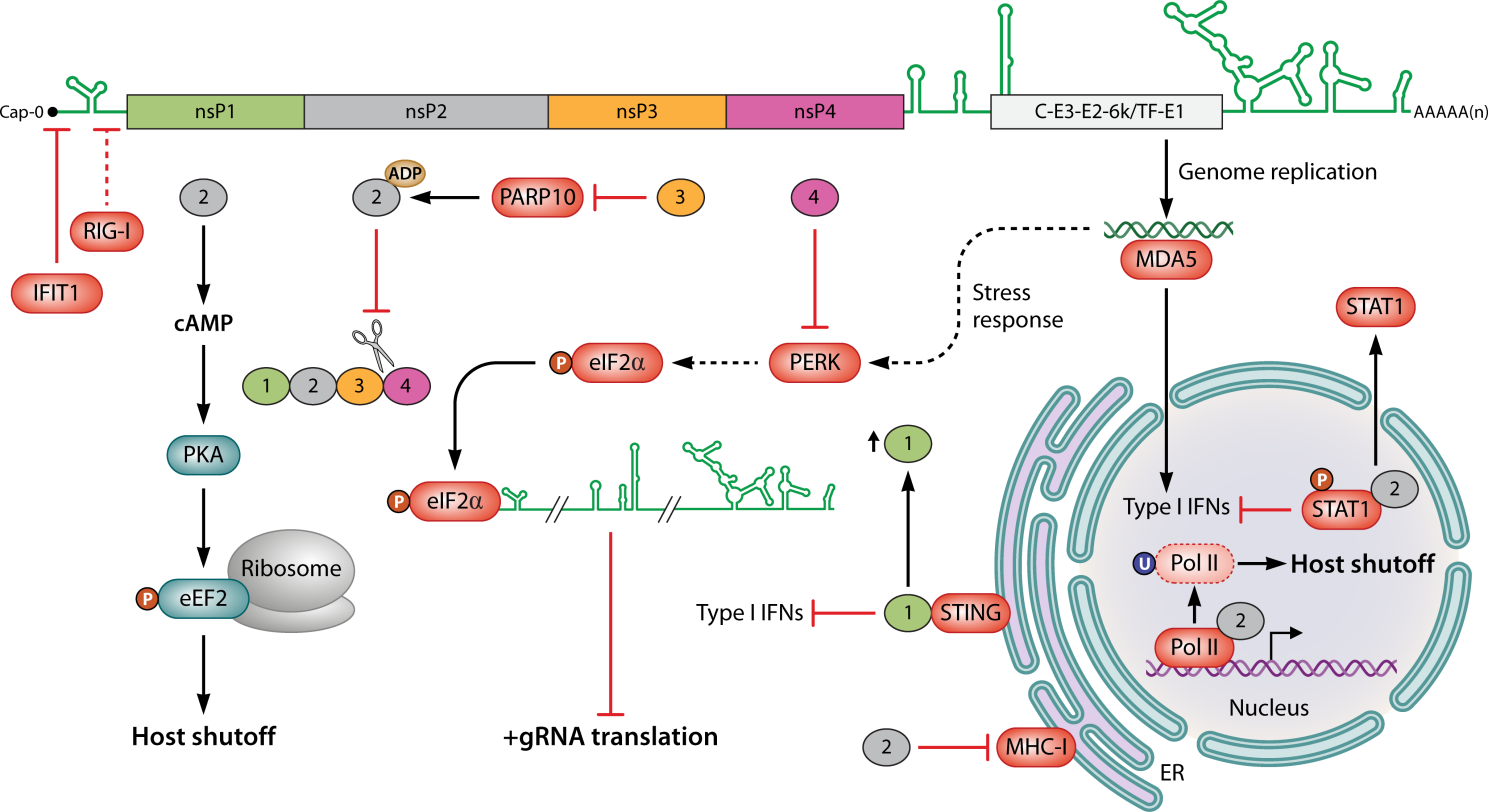
Antiviral host interactions and viral antagonism during alphavirus replication. Alphavirus infection activates RNA sensors RIG-I and MDA5, leading to type I interferon (IFN) production. Downstream IFN-stimulated genes (ISGs), such as IFIT1, restrict cap-0-dependent translation of viral RNAs. In parallel, cellular stress responses triggered during replication activate the PERK–eIF2α pathway, suppressing translation of genomic RNA (+gRNA). Mono-ADP-ribosylation of nsP2 by PARP10 inhibits its protease activity. To overcome these restrictions, alphavirus nsPs deploy multiple antagonistic mechanisms. The extreme 5′ terminus of +gRNA adopts a conserved stem-loop structure that functionally substitutes for cap-1 modification and limits IFIT1 binding. nsP2, a central antagonist of host responses, translocates to the nucleus to promote ubiquitin-dependent degradation of RNA polymerase II (Pol II) and nuclear export of STAT1, thereby inhibiting IFN signaling. In both Old and New World viruses, nsP2 elevates intracellular cAMP, activating protein kinase A (PKA) and promoting eEF2 phosphorylation to suppress host translation. nsP4 counteracts PERK-mediated translational arrest to sustain viral protein synthesis, while the hydrolase activity of the nsP3 macrodomain reverses PARP10-mediated ADP-ribosylation of nsP2. In addition, CHIKV nsP1 interacts with STING at the endoplasmic reticulum (ER) to suppress cGAS-mediated IFN induction and stabilize nsP1. CHIKV nsP2 impairs antigen presentation by downregulating peptide loading onto major histocompatibility complex class I (MHC-I) molecules. P, phosphorylation; U, ubiquitination.

Recent studies further indicate that alphavirus replicases, particularly those of SFV and SINV, can generate immunostimulatory dsRNA when expressed in isolation, including species derived from host RNA templates. These RNA byproducts are potent activators of RIG-I and MDA5 in immunocompetent cells (38, 39). Thus, although replication organelles provide partial immune shielding, efficient replication requires active modulation of innate immune signaling pathways. For example, CHIKV nsP1 directly engages a cytosolic loop of STING, dampening cGAS-mediated IFN signaling while stabilizing nsP1 expression in infected cells (40) (Fig. 2).

In addition to its role in spherule formation, nsP1 possesses methyltransferase and guanylyltransferase activities that, together with the nsP2 triphosphatase function, generate a cap-0 structure on positive-sense viral RNAs (41–44). Of note, a fraction of genomic RNAs remain uncapped. Enhanced capping efficiency, as observed for a SINV nsP1 D355A mutant, reduces viral infectivity and particle assembly through mechanisms that remain incompletely understood and appear independent of RNA synthesis (45–47). Recent work has further revealed that nsP1, despite lacking canonical Nudix hydrolase motifs or a phosphatase domain, exhibits de-capping activity toward viral RNAs mediated by reversible guanylyl transfer. CHIKV nsP1 can also remove 5′ caps from host mRNAs, suggesting a direct role in host translational shutoff (48, 49).

In contrast to many positive-sense RNA viruses whose RNAs carry a cap-1 structure with 2′-O-methylation that mimics cellular mRNA, alphaviral RNAs lack this modification and are thus susceptible to sensing by RIG-I and restriction by specific IFN-stimulated genes (ISGs), particularly IFIT1 (50, 51). To counteract this, the extreme 5′ ends of genomic and subgenomic alphavirus RNAs adopt a conserved stem-loop structure that functionally substitutes for cap-1 modification by limiting IFIT1 binding and permitting efficient translation (52, 53) (Fig. 2).

nsP2 plays central roles in polyprotein processing, RNA replication, and immune evasion. Its protease activity mediates cleavage of the nonstructural polyprotein precursors, releasing nsP4 and coordinating the transition from negative- to positive-strand RNA synthesis through sequential P1|23 and P2|3 cleavage events (54, 55). The amino-terminal region of nsP2 contains a superfamily 1 helicase domain whose RNA unwinding activity requires the presence of intact carboxyl-terminal protease and methyltransferase-like (MTL) domains. These domains bind strongly to viral RNA and direct helicase-domain binding to RNA through a flexible interdomain linker region (56, 57). Disruption of conserved helicase–RNA stacking interactions, exemplified by the F287A mutation, impairs subgenomic RNA synthesis (58, 59). The coordination of polyprotein processing by nsP2 also determines the accumulation and function of specific precursor intermediates, such as P23, which serves as a critical regulator of spherule formation and viral RNA synthesis (59, 60) (Fig. 1). Structural and biochemical studies on P23 reveal an extended RNA-binding interface spanning the nsP2 protease and the nsP3 alphavirus unique domain (AUD) and that the P2|3 cleavage site is occluded *in cis*, rendering its processing highly regulated (59).

Old World nsP2 is a potent suppressor of type I IFN responses and a major determinant of cytopathogenicity (61, 62). Cleavage-defective mutants that fail to generate free nsP2 can produce near-wild-type levels of positive-strand RNA in IFN-deficient baby hamster kidney (BHK-21) cells but are strongly restricted in cells with intact type I IFN signaling, highlighting the dual replicative and immunomodulatory roles of nsP2 (63). Mechanistically, nsP2 traffics to the cell nucleus and promotes the ubiquitin-dependent proteasomal degradation of Rpb1, the catalytic subunit of RNA polymerase II, resulting in transcriptional shutoff (64) (Fig. 2). Encephalitic alphaviruses achieve host transcriptional shutoff through the capsid protein, which forms tetrameric complexes with nuclear import and export proteins to block nuclear pores and impede the trafficking of host transcriptional factors (65).

As part of the host antiviral response, ADP-ribosylation of nsP2 by host poly(ADP-ribose) polymerase PARP10 suppresses its protease activity, thereby disrupting polyprotein processing and impairing viral replication (66–68). However, this restriction is counteracted by the N-terminal macrodomain of nsP3, which possesses ADP-ribose hydrolase activity (Fig. 2). Consistent with this, mutations that reduce macrodomain hydrolase function in CHIKV nsP3 impair replicase activity and virus viability in proportion to the degree of enzymatic loss, although it remains unclear whether these defects arise solely from impaired nsP2 function or reflect broader effects on host ADP-ribosylated targets involved in replication (66, 69). In addition, the MTL domain of CHIKV nsP2 is implicated in downregulating peptide loading onto MHC class I via an unknown mechanism, thereby promoting viral persistence in joint and muscle tissues (70) (Fig. 2).

## HOST INTERACTIONS MEDIATED BY NSP3 ARE HIGHLY DIVERGENT

The precise role of nsP3 in alphavirus replication remains incompletely understood, particularly due to its modular architecture and extensive functional plasticity. In addition to the essential roles of the macrodomain described above (60, 66, 69), the C-terminal hypervariable domain (HVD) serves as a hub for host protein recruitment but exhibits striking sequence divergence across the genus (71–74). This divergence is a major determinant of species- and cell type-specific host interactions and contributes to differences in tropism and pathogenesis (75, 76). For example, a CHIKV chimera expressing nsP3 from its closest relative, o’nyong-nyong virus (ONNV), enabled productive infection of *Anopheles* mosquitoes, a natural vector of ONNV, demonstrating that nsP3 is a critical determinant of vector specificity (77). The HVDs of both Old World and New World nsP3 sequester distinct components of stress granules via conserved short linear motifs that bind G3BP1/2 in Old World viruses and FXR family proteins (FXR1/2 and FMR1) in New World viruses (78–80) (Fig. 1B). In CHIKV and Semliki Forest virus (SFV), duplicated FGDF motifs (e.g., _449/466_L/ITFGDFD_455/472_) constitute the primary G3BP-binding sites, with an additional arginine-rich region (_398_PVAPPRRRR_406_) proposed to contribute to this interaction (80, 81). Genetic ablation of G3BP is lethal for CHIKV and strongly attenuates SFV, indicating that these interactions are not merely antagonistic to stress granule formation but are essential for efficient viral RNA replication (82, 83). Additional proviral HVD interactors include amphiphysin-1/2, which bind proline-rich SH3 motifs in the nsP3 HVD of SFV, SINV, and CHIKV (84), and the cytoskeletal adaptors CD2AP and SH3KBP1, recruited by HVDs of multiple Old World and New World alphaviruses to organize the replication complex (85). The nsP3 HVD of CHIKV also engages the RNA helicase DHX9, which enhances early nonstructural protein translation but is later selectively degraded, relieving inhibition of RNA synthesis and promoting the transition from translation to genome replication (86).

nsP3 also engages tissue-specific host factors to shape tropism and pathogenesis. Interaction with four-and-a-half LIM domain protein 1 (FHL1), a regulator of myocyte differentiation, is critical for CHIKV and ONNV replication within muscle and joint tissues (87–89) (Fig. 1B). Notably, the West African genotype of CHIKV, which is associated primarily with enzootic transmission, exhibits reduced FHL1 binding compared with the more virulent Asian and East/Central/South African lineages, linking strain-specific nsP3-host interactions to pathogenicity (88). Conversely, FHL1 is not required for other arthritogenic viruses such as Mayaro virus (MAYV) and Ross River virus (RRV). Both viruses caused wild-type-like disease in *Fhl1^−/−^* C57BL/6 mice (89). More broadly, the extreme sequence variability of the HVD permits tolerance of mutations and large deletions, as exemplified by the successful experimental insertion of fluorescent reporter genes and by the 62-amino-acid C-terminal truncation that constitutes the principal attenuating lesion in the live-attenuated IXCHIQ vaccine strain of CHIKV (90–92). Additional motifs within the HVD, such as a conserved YXXM sequence, have been implicated in metabolic reprogramming and enhanced virulence of RRV (93).

The central region of nsP3, termed the AUD, is structurally conserved and oligomerizes into a helical scaffold at the spherule neck juxtaposed to the nsP1 pore (Fig. 1B). This structure associates with nascent RNA and may direct genomic and subgenomic RNAs toward translation or virion assembly (94, 95). The AUD contains a cysteine-coordinated zinc-binding motif. Mutations within this motif, as well as in other conserved residues, impair or abolish viral replication. Whether these defects arise from disruption of helical scaffold assembly itself, however, remains unclear (96).

The opal stop codon at the nsP3|4 junction regulates the P123/P1234 ratio and is essential for proper polyprotein processing and spherule stability in vertebrate cells. In addition, it contributes to evasion of antiviral RNA interference in mosquito vectors (76, 97–100). Replacement of the opal codon with amber or ochre stop codons imposes more stringent translational termination and reduces readthrough efficiency at 37°C. Conversely, substitution with sense codons promotes polyprotein processing and attenuates viral replication in vertebrate cells, whereas such modifications are generally better tolerated in mosquito cells (101). Circulating SFV and ONNV strains naturally encode arginine or cysteine in place of the opal stop codon, a feature proposed to favor controlled replication in mosquitoes and potentially limit their pandemic potential in humans (102).

Finally, post-translational modification of nsP3 also contributes to its multifunctionality. In VEEV, IKKβ-mediated phosphorylation of nsP3 has been shown to promote negative-strand RNA synthesis and virion assembly (103, 104) (Fig. 1B).

## NSP4 STABILITY AND REPLICATION FIDELITY

The viral RdRp nsP4 is inactive and highly unstable when expressed in the absence of P123, owing to a conserved N-terminal tyrosine that targets the protein for ubiquitin-dependent N-end rule degradation (105–108). Substitution of this residue with a primary methionine (Y1M) or with aromatic or histidine residues stabilizes nsP4 but attenuates replication. Except for its first ~100 amino acids, nsP4 is the most conserved component of the alphavirus replicase and is functionally interchangeable across the genus. Many replicase chimeras composed of the parental P123 precursor and heterologous nsP4, including that from the insect-specific Eilat virus, remain competent for viral RNA synthesis (109). In addition to its polymerase activity, nsP4 exhibits terminal adenylyltransferase activity, suggesting a role in poly(A) tail synthesis and repair (110).

Mutations that target nsP4 fidelity modulate viral replication dynamics. A C483G substitution reduces polymerase fidelity and increases mutagenesis and template switching, thereby promoting the accumulation of truncated, defective interfering (DI) genomes (111, 112). Conversely, the C483Y substitution has been proposed to generate a high-fidelity variant of CHIKV nsP4 that maintains near-wild-type levels of genomic RNA synthesis but displays context-dependent attenuation *in vivo*. In susceptible neonatal mice infected subcutaneously, the C483Y mutant virus caused reduced disease, presumably reflecting stronger immune control of a more genetically constrained viral quasispecies (113). However, immunocompetent adult mice challenged in the footpad with the same mutant virus unexpectedly developed more severe arthritis and myalgia. These discordant outcomes have led subsequent studies to question whether C483Y reliably confers a stable high-fidelity phenotype (114, 115).

## HISTORY OF ALPHAVIRUS-DERIVED EXPRESSION SYSTEMS

Conserved sequence elements (CSEs) refer to the conserved features of the viral genome that are essential for RNA replication, transcription, packaging, and assembly. Modern recombinant alphavirus replicons exploit these elements in the context of native viral genomes and replace the structural gene cassette with a heterologous gene of interest (GOI) expressed under control of the 26S subgenomic promoter. These systems retain the ability to form membrane-associated replication complexes and to generate (sub)genomic RNAs, but are incapable of producing infectious virus particles because they lack the essential structural proteins.

**Fig 3:**
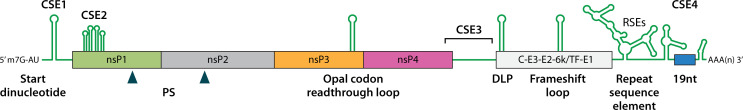
Conserved sequence elements and regulatory RNA structures in alphavirus genomes. Viral RNA replication is governed by four conserved sequence elements (CSEs) distributed along the genome. At the 5′ end, CSE1 forms a stem-loop structure that functions as the promoter for the synthesis of full-length genomic RNA from the negative-strand template in coordination with CSE2. Immediately downstream, the 51-nt CSE2 within the nsP1 coding region supports efficient positive- and negative-strand RNA synthesis. The subgenomic promoter (CSE3), spanning from the C-terminus of nsP4 to the intergenic region (−98 to + 14 in SINV), directs transcription of subgenomic RNA. At the 3′ end, CSE4, a conserved 19-nt AU-rich sequence (AUUUUGUUUUUAAUAUUU) located immediately upstream of the poly(A) tail, acts as the promoter for antigenome synthesis, likely through long-range RNA interactions with CSE2. Several additional RNA elements contribute to viral replication and gene expression. Packaging signal loops (PS; arrowheads), located within nsP1 or nsP2 coding regions depending on the virus, are recognized by the capsid protein to ensure selective incorporation of full-length genomic RNA into virions. The nsP3|4 junction frequently contains an opal stop codon (UGA) embedded within a stem-loop structure that regulates readthrough and production of the P1234 polyprotein. In SINV and SFV, a translation-enhancing downstream loop (DLP) within the N-terminal 34 amino acids of capsid promotes efficient subgenomic RNA translation. A −1 ribosomal frameshifting element within the structural region drives expression of the transframe protein (TF). Repeated sequence elements (RSEs) within the 3′ UTR vary in number among alphaviruses and influence RNA stability, replication efficiency, and host adaptation.

The conceptual basis of alphavirus replicon technology emerged from early studies of DI genomes that sought to identify the minimal CSEs required for replication and packaging. When structural proteins are supplied *in trans*, DI RNAs or full-length replicons can be selectively encapsidated into single-cycle viral replicon particles (VRPs). Early recombinant alphavirus expression systems were developed using SINV DI genomes. In its first published use, a ~2.2 kb DI genome was cloned into a plasmid under the control of an SP6 bacteriophage promoter. This cDNA was transcribed and delivered into chicken embryonic fibroblasts together with a replication-incompetent SINV helper virus (116). Systematic deletions in the DI cDNA subsequently defined a minimal set of essential CSEs, including a 162-nt region at the 5′ terminus comprising the 5′ untranslated region (CSE1) and a 51-nt stem-loop-rich region within nsP1 (CSE2), as well as a 19-nt AU-rich region from the 3′ end of the genome (CSE4). Together with the 26S subgenomic promoter (CSE3), these elements were shown to be collectively required for efficient RNA amplification and production of infectious particles (117) (Fig. 3).

The substitution of native viral sequences from a functional DI genome with a heterologous reporter gene (chloramphenicol acetyltransferase, CAT) demonstrated that these elements were sufficient to drive foreign gene expression, establishing the core design principles of alphavirus replicon vectors (118). Breakthroughs in cloning the entire SINV genome enabled the construction of the first full-length alphavirus replicon system, which achieved robust CAT expression under control of the viral 26S subgenomic promoter across a wide range of vertebrate and invertebrate cells (119, 120). However, early systems were limited by low transfection efficiency and recombination between replicon and helper RNAs (121), which were later alleviated by electroporation-based transfection and by bipartite helper systems in which capsid and glycoprotein genes were expressed from separate RNAs to minimize recombination (122–124).

Further refinements stemmed from the observation that the N-terminal 30–34 amino acids of the SINV and SFV capsid proteins function as translational enhancers for subgenomic RNA expression (124–126) (Fig. 3). Incorporation of this enhancer upstream of the SFV glycoprotein cassette, linked through a foot-and-mouth disease virus ribosomal skipping 2A peptide, enabled native-like expression levels of the separated structural proteins in helper systems (124). Similarly, in-frame N-terminal fusion of full-length capsid to a GOI enhanced fluorescent reporter expression in SINV and CHIKV backbones (127). Importantly, the intrinsic C-terminal autoproteolytic activity of capsid ensures efficient release of the downstream protein, thereby preserving proper folding and intracellular localization of the GOI product.

Subsequent studies using VRPs elucidated the molecular basis of alphavirus RNA packaging. It was found that capsid selectively recognizes conserved stem-loop structures containing GGG trinucleotide motifs, which are distributed within the nsP1 (e.g., SINV, VEEV) or nsP2 (e.g., CHIKV, SFV) coding regions rather than being confined to a single, discrete packaging signal (128–130). Importantly, capsid can indiscriminately recognize GGG-containing stem-loops for packaging. Chimeric alphaviral RNAs encoding VEEV nsPs can be encapsidated by CHIKV capsid proteins to produce viable, albeit attenuated, viruses (131). Such chimeric viruses represent promising candidates for the development of live-attenuated vaccines.

Given the malleability of the alphavirus genome and the naturally low virulence of SINV in humans, dual-subgenomic promoter viruses had been proposed as candidate live-attenuated vaccines. The duplication of the 26S promoter within full-length viral genomes enabled simultaneous expression of structural proteins and a GOI from two subgenomic RNA molecules (132). A shift to New World dual-promoter systems emerged upon the observation that the more pathogenic VEEV is naturally lymphotropic in C57BL/6 mice and Syrian golden hamsters via efficient infection of dermal dendritic cells that traffic to draining lymph nodes (133, 134). The construction of avirulent derivatives for dual-promoter vaccine design leveraged targeted glycoprotein point mutations that markedly reduced viremia and neurotropism while restricting replication in lymphoid tissues (135, 136). While these systems proved immunogenic and protective in animal models, they were ultimately constrained by polymerase template switching, accumulation of truncated genomes due to structural limits on packaging oversized genomic RNA, and poor nsP4 proofreading during replication of a large subgenomic payload (136, 137).

Although dual-promoter alphavirus vectors have fallen out of favor, attenuated VEEV-based platforms have maintained considerable interest as vaccine vectors. A VEE-VRP system protected BALB/c mice against influenza challenge while limiting recombination through bipartite defective-helper RNAs (123). Sequential immunization experiments demonstrated limited vector immunity, even following prior exposure to the homologous replicon backbone. Subsequent use of lymphotropic SINV bipartite helper RNAs enabled VEE-VRP production outside biosafety level 3 containment (138, 139). Both wild-type and chimeric VEE-VRPs produced higher transgene expression and cytotoxic T cell responses than reciprocal SIN-VRPs, highlighting the strength of New World alphavirus expression systems (139). Furthermore, unlike SINV and SFV systems, high-level transgene expression from VEEV replicons does not require a subgenomic capsid enhancer. Despite these advances, scalable manufacturing of alphavirus VRPs remains a practical limitation, even with the use of stable packaging cell lines (140).

## FROM CONVENTIONAL MRNA VACCINES TO SELF-AMPLIFYING RNA PLATFORMS

The COVID-19 pandemic catalyzed the first large-scale use of conventional mRNA vaccines against SARS-CoV-2. These vaccines consist of capped, *in vitro*-transcribed mRNAs encoding viral antigens that are delivered using lipid nanoparticle (LNP) formulations (141). Although highly effective at inducing protective immunity, conventional mRNA vaccines are limited by the intrinsic instability and short intracellular half-life of mRNA, which constrain the duration of antigen expression. This transient antigen production has been proposed as one factor contributing to the limited durability of immune memory and the need for periodic booster immunizations (142).

A logical extension has thus been the development of sa-mRNA platforms that encode both the antigen and a viral replicase, allowing RNA amplification and prolonged antigen expression. Alphavirus replicon systems, given their extensive studies over decades, provided the conceptual and molecular foundation for a dose-sparing approach in vaccine prophylaxis (13, 143–148). Among available backbones, VEEV replicons have emerged as the dominant platform, owing to their pronounced lymphotropism, relatively low vector immunity, strong innate immune activation, and robust heterologous gene expression compared with Old World alphaviruses such as SINV or SFV (149). Consistent with this trend, all licensed sa-mRNA vaccines (KOSTAIVE and GEMCOVAC) and publicly described alphavirus-derived sa-mRNA candidates currently in FDA clinical trials for SARS-CoV-2, influenza, or rabies employ VEEV replicase backbones (150). However, despite this progress and extensive prior work on alphavirus expression systems, key aspects of nonstructural protein function and CSEs in the context of sa-mRNA remain incompletely understood.

## HOST CYTOTOXIC AND STRESS RESPONSES ELICITED BY ALPHAVIRUS REPLICONS

Alphavirus replicons are often reported to achieve very high intracellular RNA copy numbers, although quantitative estimates vary and are frequently poorly substantiated (151–153). Regardless of amplification magnitude, replicon activity induces substantial cellular stress even in the absence of structural genes, making control of cytopathic effects (CPE) essential for sustained antigen expression, efficient antigen presentation, and reduced vaccine reactogenicity.

Most contemporary VEEV-based sa-mRNA platforms are derived from the TC-83 live-attenuated vaccine strain, generated by serial passage of the Trinidad Donkey (TRD) strain in guinea pig heart cells (154). In packaging-deficient TC-83 replicons, attenuation is largely attributed to a G-to-A substitution at position 3 of the 5′ UTR, which alters the local stem-loop secondary structure and generates a longer, more exposed 5′ overhang, thereby enhancing IFIT1-mediated restriction of translation initiation (52, 155, 156) (Fig. 2). Consistent with this, TC-83 displays virulence comparable to wild-type TRD VEEV in type I IFN receptor (IFNAR)-deficient mice, while inducing reduced CPE in IFN-primed mouse fibroblasts (157, 158). Thus, attenuation of TC-83 replicons is context-dependent and driven largely by intact type I IFN signaling.

In permissive cell lines such as BHK-21, VEEV and western equine encephalitis virus (WEEV) replicons are intrinsically less cytopathic than SINV replicons, and the TC-83 5′ UTR mutation further reduces CPE (159). Comparative studies across SINV, VEEV, and WEEV dual-promoter replicons further revealed that non-cytopathic variants can be selected through adaptive mutation, often converging on substitutions within the C-terminal MTL domain of nsP2 (160–162). A conserved proline residue at position 726 (P726) in SINV and SFV and at position 718 in the West African genotype of CHIKV appeared to be a key determinant of cytopathogenicity.

Subsequent studies demonstrated that enzymatic activities of the nsP2 MTL domain are not strictly required for CPE induction, suggesting instead a dominant role for nsP2-mediated inhibition of host transcription (61, 62). Non-cytopathic nsP2 variants often exhibit disrupted GTPase, helicase, or protease activities and can be maintained in permissive cell lines, although compensatory mutations in other nsPs are frequently required for productive replication in human cells, where isolated nsP2 mutations may otherwise be lethal (160, 162, 163). In SINV replicons, P726 is additionally implicated in host translational shutoff by interfering with PKR-dependent phosphorylation of eIF2α, and substitution of this residue (e.g., P726G) alleviates translational repression (164). Conversely, CHIKV nsP4 appears to suppress PERK-mediated eIF2α phosphorylation early in infection, thereby relieving translational inhibition and promoting viral protein synthesis (165) (Fig. 2).

Interestingly, non-cytopathic nsP2 mutations are often associated with hyperactive polyprotein processing, which reduces antigenome synthesis and thereby limits the intracellular pool of nsP2 capable of antagonizing host transcription and translation (160). Despite reduced translation of the nonstructural polyprotein upon eIF2α phosphorylation, Old World alphaviruses retain efficient transgene expression due to a stable RNA hairpin, termed the downstream loop (DLP), located within the capsid translational enhancer (Fig. 3). This element permits robust translation of subgenomic RNA in the absence of eIF2α and eIF4G and can support initiation from non-AUG start codons (166, 167). Consequently, non-cytopathic Old World replicons can sustain sufficient antigen expression for effective processing and presentation. However, the translational advantage conferred by the DLP may depend on the context of active viral replication, as subgenomic RNA delivered by replicon transfection has been observed to preferentially engage canonical translation pathways (168).

**Fig 4:**
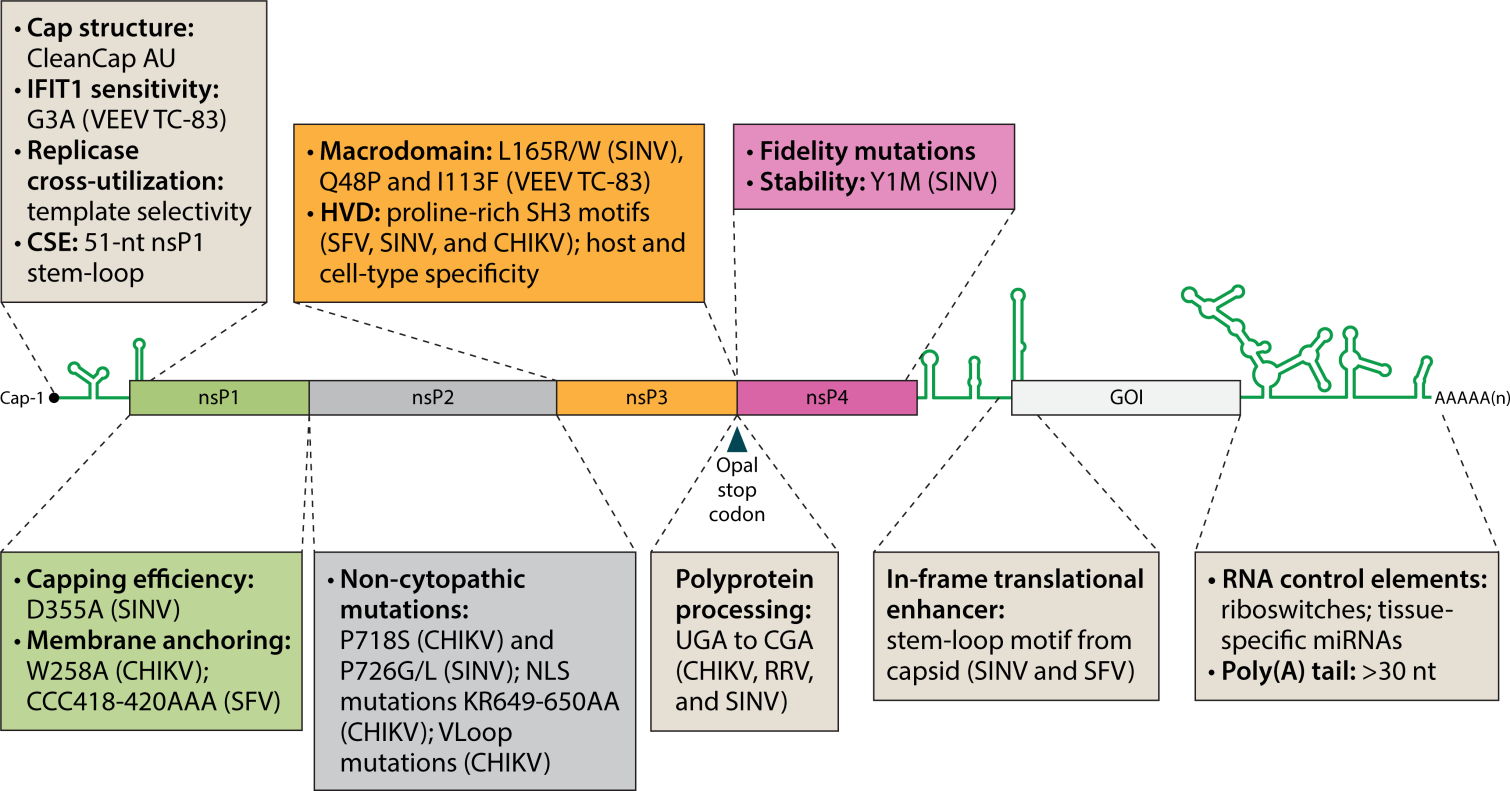
Engineering nonstructural proteins and RNA elements for optimized sa-mRNA backbones. Schematic of an alphavirus-derived sa-mRNA highlighting viral nonstructural proteins (nsP1–4) and conserved sequence elements (CSEs) that can be rationally modified to tune replication, innate immune sensing, cytotoxicity, and transgene expression. The 5′ UTR is a key regulatory hub controlling replicase specificity and the balance between IFIT1 restriction and type I IFN induction. Current VEEV TC-83-based sa-mRNA production uses CleanCap AU (cap-1) to enhance translation and mitigate IFIT1 restriction of incoming RNA, despite increased IFIT1 susceptibility associated with the G3A mutation. The 5′ UTR sequence also influences template selection by heterologous replicases, an emerging strategy in *trans*-amplifying RNA systems. Mutations that destabilize the 51-nt CSE2 within nsP1 can reduce RNA replication and alleviate cytotoxicity. Likewise, nsP1 variants that enhance capping efficiency or weaken membrane anchoring can modulate replication output and cellular stress. Targeting residues in Old World nsP2 associated with cytopathogenicity can relieve host shutoff and attenuate cytopathic effects. Mutations in the nsP3 macrodomain can shift the balance between genomic replication and subgenomic RNA transcription, potentially enhancing transgene expression, while modifications in the nsP3 hypervariable domain (HVD) can alter host factor recruitment to reduce cytopathogenicity or enable cell- and tissue-specific replication. Variants of nsP4 that alter polymerase stability or fidelity modulate RNA amplification, mutation rates, and DI RNA formation, thereby affecting cytotoxicity, transgene integrity, and expression durability. Replacement of the opal stop codon at the nsP3|4 junction with a sense codon can attenuate replication in vertebrate cells of certain alphaviruses. Incorporation of the capsid-derived translational enhancer sequence into the transgene can increase expression in Old World backbones. Finally, regulatory modules within the 3′ UTR, including riboswitches, tissue-specific microRNA target sites, and optimized poly(A) tail length, can be used to fine-tune expression kinetics and tissue tropism.

Given the divergent host shutoff strategies employed by Old World and New World alphaviruses, targeting nsP2 nuclear translocation has emerged as a means to partially restore innate immune signaling and reduce cytopathogenicity for Old World viruses, although these effects can be highly virus- and strain-dependent (169). For example, mutation of the nsP2 nuclear localization signal (NLS) in SFV enhanced type I IFN responses and reduced cytotoxicity when combined with an additional P718T substitution (163, 170). Mutations at conserved proline residues (P718 in CHIKV or P726 in SINV) or within the NLS (e.g., KR649-650AA) improved nuclear import of phosphorylated STAT1 and restored functional IFNAR signaling (171–173) (Fig. 4). It is noted that putative NLS mutations at analogous positions do not necessarily confer a non-cytopathic phenotype across different alphavirus species, or even among strains of the same virus (162, 174). Later work on developing non-cytopathic Old World replicons identified a weakly conserved peptide motif (called “VLoop”) within the nsP2 MTL domain, whose mutations reduced nsP2 nuclear localization and subsequent host transcriptional arrest (Fig. 4). These mutations conferred additional attenuation by impairing polyprotein processing in CHIKV, but not in SINV (37, 175). In parallel, a PKR-independent mechanism of translational shutoff has been proposed, in which the nsP2 helicase domain of CHIKV and VEEV elevates intracellular cAMP levels, thereby activating protein kinase A and downstream phosphorylation of EEF2 (176) (Fig. 2).

By contrast, replicons derived from New World alphaviruses such as VEEV generally require fewer genetic modifications to achieve acceptable cytopathic profiles, making them more practical backbones for sa-mRNA applications. Nevertheless, nsP2-mediated translational shutoff is conserved among New World alphaviruses, and the C-terminal region of nsP2 remains a key barrier to further attenuation and optimization (177). *In vitro* evolution of VEEV TC-83 sa-mRNA under exogenous type I IFN pressure selected mutations in the nsP3 macrodomain that reduce its hydrolase activity and slow replication kinetics (178) (Fig. 4). Although the mechanisms are not fully resolved, these variants exhibit reduced innate immune activation and cytotoxicity with enhanced transgene expression (178), indicating that optimal sa-mRNA performance depends on a finely tuned balance among replicase activity, innate immune sensing, and viral antagonism rather than on maximal RNA amplification alone.

## CONSERVED SEQUENCE ELEMENTS TUNE SA-MRNA REPLICATION AND EXPRESSION

Alphavirus CSEs also provide powerful levers for tuning replicon performance. In addition to enhancing IFIT1-mediated restriction, the G3A mutation in the 5′ UTR of VEEV TC-83 alters competition between the genomic and subgenomic promoters for viral replicase components, thereby reducing subgenomic RNA synthesis (155). The conserved 51-nt element (CSE2) within nsP1, present across alphaviruses, is essential for positive-strand RNA synthesis and functions in concert with the 5′ UTR (179, 180). Consistent with this, and drawing on DI genome architecture, insertion of a 228-nt sequence encompassing the genomic 5′ UTR and CSE2 downstream of the VEEV subgenomic promoter markedly enhances transgene expression across multiple mammalian cell types (181). To prevent fusion of the GOI to the nsP1-derived peptide encoded by CSE2, the design further incorporates self-processing elements, such as a 2A peptide, ubiquitin, or 2A in combination with an NLS-deficient, non-cytopathic capsid sequence, positioned upstream of the transgene (181). These findings delineate alternative engineering strategies for New World alphavirus-based sa-mRNA and VRP platforms.

Substantial sequence divergence within the nsP3 HVD and the 3′ UTR reflects natural mechanisms for tuning host interactions and RNA stability (Fig. 4). Viruses in the VEE serogroup harbor relatively short 3′ UTRs (100–200 nt), whereas WEE-complex and SFV-complex viruses contain longer regions of ~200–300 nt and ~300–600 nt, respectively (182, 183). Repeat sequence elements (RSEs) within the 3′ UTR enhance RNA stability in vertebrate cells by recruiting host RNA-binding proteins such as Human antigen R (HuR), which suppresses poly(A) shortening and exonucleolytic decay while also promoting efficient replication in mosquito vectors (184–187) (Fig. 3). From an evolutionary standpoint, recombination events that lengthen the 3′ UTR have been proposed to enhance adaptation of alphaviruses to mosquito vectors during transitions from aquatic hosts and, more recently, to stabilize transmission bottlenecks of epidemic CHIKV strains in *Aedes* mosquitoes (188, 189). The presence of additional RSEs that exert neutral effects in SINV but detrimental effects in CHIKV on viral replication in mammalian cells warrants further investigation into the balance required for optimal fitness across vertebrate and invertebrate hosts (189, 190). The length and composition of the poly(A) tail further regulate antigenome synthesis. For example, antigenome production in SINV is reduced when the poly(A) tail is shorter than 25 residues and is completely abolished when it falls below 11 residues (191). Consistent with a role for 3′ end architecture in host adaptation, VEEV TC-83 chimeras bearing heterologous 3′ UTRs from epizootic and enzootic subtypes exhibited differential restriction by ISGs, such as IFIT2 (192).

## ENGINEERING RNA CHEMISTRY AND ARCHITECTURE TO OPTIMIZE SA-MRNA PERFORMANCE

Chemical and structural optimization of the RNA template has become a central strategy in sa-mRNA engineering for balancing robust replicase activity with controlled innate immune activation (144). Systematic screening has identified nucleotide modifications compatible with alphavirus RNA replication, including 5-hydroxymethylcytidine (hm5C), 5-methylcytidine (m5C), and 5-methyluridine (m5U), which attenuate innate sensing while preserving RdRp function and efficient RNA amplification (193).

The architecture of the poly(A) tail has also been explored as a determinant of RNA stability and translation efficiency. Conventional non-replicating mRNA vaccines are constructed with 60–120 nt poly(A) tails, similar in length to those of SINV and SFV; however, alphavirus negative-strand RNA synthesis is not further improved by increasing tail length from 25 to 34 adenosines (191, 194, 195). Thus, extension of sa-mRNA poly(A) tails beyond the minimally sufficient 25 nucleotides may not be a strong determinant of payload expression.

Current sa-mRNA manufacturing further employs cap-1 analogs (e.g., CleanCap AU) to enhance translation and dampen innate sensing during the initial launch phase (144, 150). However, following replication, newly synthesized viral RNAs acquire the natural cap-0 structure and 5′ triphosphate intermediates generated by nsP1 and nsP2, rendering cap-1 modification transient and largely restricted to the input RNA population.

*Trans*-amplifying RNA (taRNA) platforms are bipartite systems that reduce manufacturing barriers associated with encapsulating long sa-mRNA transcripts. In this approach, the replicase is encoded within a non-replicating conventional mRNA, whereas the *trans*-replicon (TR) adopts a DI-like architecture containing all essential alphaviral CSEs required for self-amplification of the GOI (196). Recent simplification of this design, achieved by removing CSE3 and eliminating all CSE1/2-localized AUG start codons upstream of the GOI start site, further enhanced RNA replication and transgene expression (197).

Notably, taRNA systems can exploit the partial cross-compatibility of alphavirus replicase-template pairs, with SFV replicase supporting highly efficient amplification of heterologous SINV-derived TRs in human cells (197, 198). *In vitro* evolution of such hybrid systems in the presence of competing TRs from different alphavirus species selected variants with enhanced replication kinetics and transgene expression, such as SFV-derived mutants bearing a short 5′ extension (AUAAAA(A)), previously linked to efficient negative-strand initiation (197, 199). These findings highlight the 5′ UTR as a critical regulatory hub that warrants continued investigation (Fig. 4), both for its role in governing replicase-template specificity and for its involvement in balancing IFIT1-mediated restriction and type I IFN induction during alphavirus infection (53, 198).

## BIODISTRIBUTION AND BIOSAFETY CONSIDERATIONS FOR SA-MRNA PLATFORMS

LNP-mRNA vaccines are typically administered intramuscularly, where immune activation arises from antigen expression in muscle and antigen-presenting cells or from LNP drainage to lymph nodes (200). Although LNP composition and size can be tuned to restrict biodistribution, low-level accumulation in off-target tissues, including the liver and spleen, is common (201–203). Given the self-replicative capacity of alphavirus backbones, careful evaluation of *in vivo* biodistribution remains essential, as even minimal dissemination to sensitive tissues such as the central nervous system (CNS) could be detrimental (204).

To date, sa-mRNA has not been detected in the brains of vaccinated mice, possibly reflecting limited penetration of the blood-brain barrier (BBB) by the large LNP-formulated RNA cargo, although studies addressing this issue remain limited (205, 206). Nevertheless, alphavirus biology suggests additional complexity. Infectious virus-like vesicles (VLV) can be generated in the absence of capsid proteins. Here, genomic, subgenomic, or DI RNA may be nonspecifically packaged when in proximity to heterologous viral glycoproteins that drive vesicular budding from the cell surface by a poorly defined mechanism (207, 208). Such VLVs displaying foreign glycoproteins can be propagated in IFN-deficient cells and have been explored as vaccine candidates (209, 210). Although VLVs appear non-pathogenic in immunocompetent animals, their potential emergence following sa-mRNA administration and its impact on biodistribution remain poorly understood.

In addition to vesicle-mediated dissemination, direct cell-to-cell RNA transmission may further alter biodistribution. Old World alphaviruses (SFV, SINV, CHIKV), and to a lesser extent VEEV, remodel the actin cytoskeleton through nsP1 to induce filopodia-like extensions that facilitate transfer of infectious entities independently of nucleocapsid assembly (211–214). Mutations that disrupt nsP1 palmitoylation or membrane-associated hydrophobic residues reduce filopodia formation and thus represent useful engineering targets for future sa-mRNA design (33, 215, 216).

Consistent with these dissemination mechanisms, multiple, non-exclusive routes of alphavirus CNS invasion have been proposed, including neuronal transport, inflammation-induced BBB disruption, and vesicular transcytosis across endothelial cells. Glycoprotein determinants, particularly within E2, modulate neurotropism, raising theoretical biosafety considerations for sa-mRNA encoding foreign glycoproteins with CNS-targeting potential. Although such risks remain speculative, they warrant continued mechanistic investigation (217–222).

Genetic recombination between sa-mRNA and circulating alphaviruses is also theoretically possible, although superinfection exclusion is expected to strongly limit the generation of infectious chimeras (223, 224). In Old World alphaviruses, exclusion is mediated in part by premature nsP2-driven polyprotein processing, whereas in New World viruses, it involves nsP3 HVD-dependent sequestration of host factors required for RNA synthesis (225–227).

To improve controllability and safety, drug-inducible sa-mRNAs have been developed (228). Riboswitches inserted into the 5′ and 3′ UTRs of the subgenomic RNA region of VEEV TC-83 confer ligand-dependent regulation in DNA-launched and VRP-packaged replicons (229), although this strategy has yet to be applied to LNP-delivered sa-mRNAs. More broadly, spatial control of RNA distribution remains a central requirement for safe and effective gene therapy. A panel of alphavirus sa-mRNAs encapsulated in the SM-102 LNP formulation (used in the Moderna COVID-19 vaccine) exhibited tissue-specific accumulation patterns in murine organs following intramuscular or intravenous administration (230). Accordingly, incorporation of 3′ UTR microRNA target sites that restrict productive replication in undesired tissues represents a practical strategy to improve targeting precision and reduce off-target effects (231, 232) (Fig. 4).

Although VEEV remains the most widely used sa-mRNA backbone, alternative alphavirus replicons are desirable to mitigate anti-backbone immunity and to support repeated or heterologous boosting (233, 234). Old World alphaviruses, with their intrinsic myotropism, warrant renewed consideration, although additional engineering will be required to achieve expression levels comparable to VEEV-based systems (127, 230, 235). Exploration of diverse backbones may also reveal conserved T-cell epitopes that could be harnessed to elicit dual immunity against both alphaviruses and the encoded pathogen, thereby broadening the protective scope of sa-mRNA vaccine platforms (236).

## CANCER AND GENE THERAPY APPLICATIONS OF SA-MRNA

**Fig 5:**
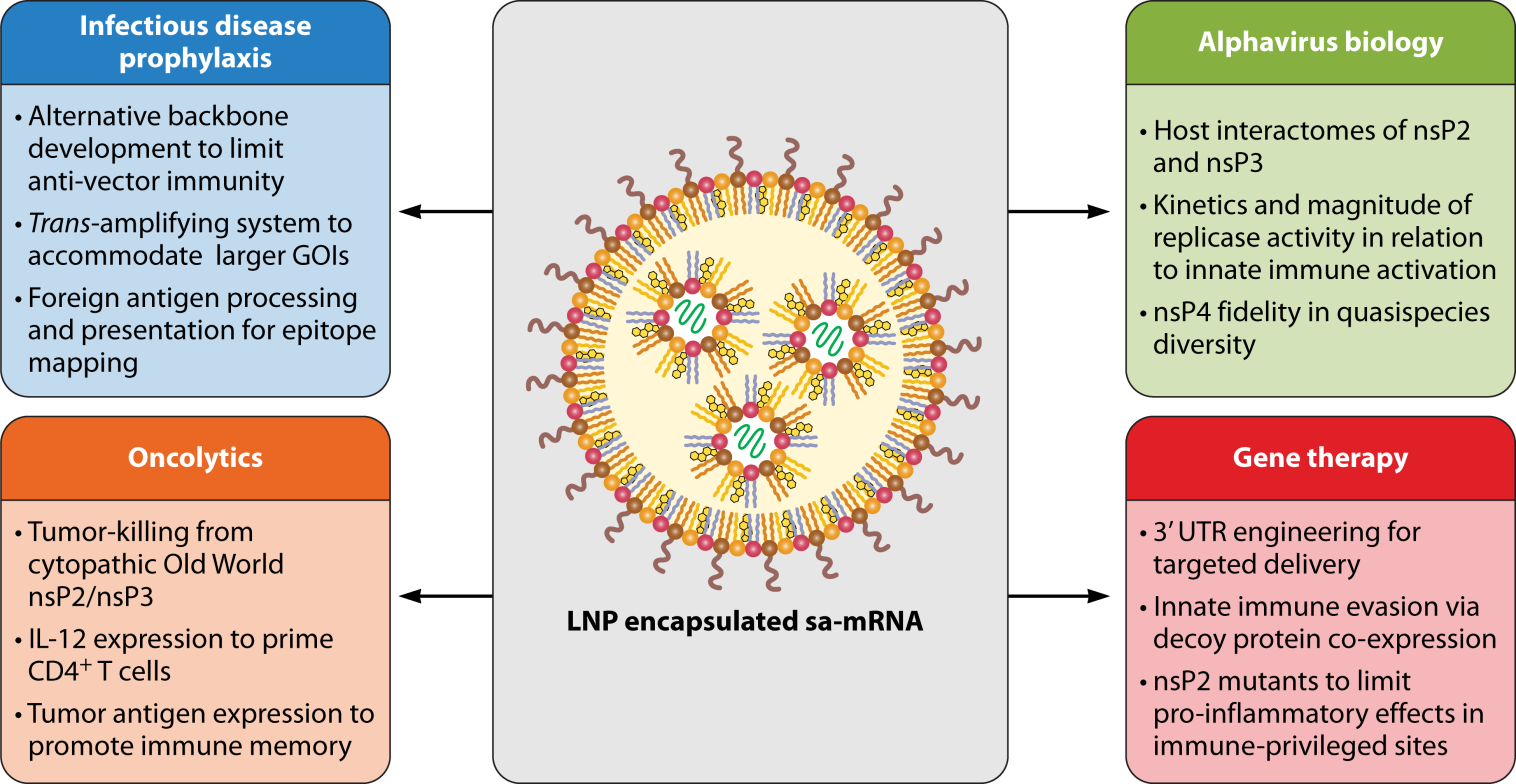
Emerging applications for next-generation sa-mRNA systems. The inherent plasticity of the alphavirus genome enables deployment of sa-mRNA platforms across diverse biomedical applications. Expanded use of sa-mRNA for infectious disease prophylaxis should incorporate a broader repertoire of alphavirus backbones to mitigate anti-backbone immunity and enable repeated or heterologous boosting. These alternative backbones may also support the development of *trans*-amplifying RNA systems capable of accommodating larger payloads, thereby overcoming current limitations in long RNA production and encapsulation. A deeper understanding of antigen processing and presentation *in vivo* will clarify how sa-mRNA platforms differ from conventional vaccines in shaping immunogenicity and protective efficacy. Oncolytic applications of sa-mRNA involve direct intratumoral administration, where wild-type Old World backbones may be advantageous due to their intrinsic cytotoxicity. Transient expression of cytokines such as interleukin-12 (IL-12) or tumor-associated antigens using arthritogenic backbones can remodel the tumor microenvironment to promote pro-inflammatory responses and overcome immune tolerance or T cell exhaustion. For gene replacement therapies, targeted delivery of sa-mRNA to specific tissues offers a strategy for sustained therapeutic protein expression. Off-target effects can be minimized through engineering of the 3′ UTR, while replication-associated innate immune activation may be mitigated by co-expression of IFN antagonists. Non-cytopathic nsP2 variants may further enable safer expression in immune-privileged sites such as the eye. Beyond therapeutic applications, alphavirus-based sa-mRNA systems provide powerful experimental platforms for probing fundamental aspects of alphavirus biology, including the functions of nsP2 and nsP3, regulation of replicase activity in the context of innate immune responses, and the role of nsP4 fidelity in viral diversity and precision control of transgene expression.

Beyond their application in prophylaxis against infectious diseases, which has been reviewed extensively elsewhere (150), sa-mRNA platforms have been actively explored for cancer immunotherapy and protein replacement applications (Fig. 5). Intramuscular delivery of a single dose of sa-mRNA expressing a chimeric human papillomavirus (HPV) and herpes simplex virus antigen generated a robust cytotoxic T cell response that reduced HPV-associated tumor burden and established a durable memory T cell population capable of preventing subsequent tumor development (237). In mouse-derived dendritic cells, however, HPV antigen expression from sa-mRNA was lower than that observed with modified non-replicating mRNA containing N1-methylpseudouridine, suggesting that (i) more cytopathic or IFN-suppressive alphavirus backbones may be advantageous in certain cancer treatment regimens, and (ii) management of double-stranded RNA intermediates remains a critical determinant of sa-mRNA vaccine performance. While the incorporation of modified bases in self-replicating systems can facilitate evasion of Toll-like receptor sensing, lower and more sustained antigen expression kinetics may, in some contexts, be preferable to the metabolic stress associated with excessive or prolonged immunogen production.

Conventional mRNA platforms have also been used to deliver cytokine adjuvants, such as interleukin-12 (IL-12), to expand and prime CD8^+^ T cell pools against melanoma and related malignancies (238, 239). An EEEV-derived sa-mRNA expressing IL-12 and IL-1 receptor antagonist induced superior antitumor responses compared to a VEEV-based backbone (240). Additional cytokines, including IL-15 and IL-18, have been evaluated for their capacity to promote natural killer (NK) cell activation and Th1-skewed immunity in preclinical models, alongside granulocyte-macrophage colony-stimulating factor (GM-CSF), which supports the differentiation and activation of monocytes into pro-inflammatory macrophages (241). Furthermore, replicon particle systems derived from VEEV, SFV, and SINV have entered clinical trials for adenocarcinomas, prostate cancer, and checkpoint inhibitor-based immunotherapy, generally demonstrating acceptable tolerability with adverse events limited primarily to fever and mild injection-site reactions (242). Despite these advances, anti-vector immunity remains a concern for these platforms, and efficient delivery and targeting of LNP-encapsulated sa-mRNA to tumor-resident dendritic cells continue to be key translational barriers. In this context, filamentous liposomal delivery systems have shown promising delivery efficiency for SFV plasmid DNA vectors encoding tumor suppressor and immune checkpoint targets such as p53 and PD-L1 (243).

The development of RNA therapeutics for protein replacement has similarly provided a framework for evaluating appropriate sa-mRNA dosing in immunocompetent but anatomically privileged tissues. In the context of retinal disease, one study showed a minimal threshold for detectable transgene expression in ocular cell lines, while higher doses were paradoxically associated with reduced exogenous protein production, suggesting a delicate balance between GOI expression and innate immune activation (244). Future studies should more systematically evaluate the contribution of regulatory RNA elements, such as the Old World virus DLP, to enhancing payload expression relative to innate immune restriction in physiologically relevant cell systems. Incorporation of innate immune antagonists or decoy proteins, such as vaccinia virus B19 or nodavirus B2, which suppress sensing of alphaviral dsRNA, may help mitigate dose-dependent inflammatory responses and improve the safety profile of sa-mRNA therapeutics (244, 245). Encouragingly, a recent study showed that intramuscular delivery of sa-mRNA encoding the *Nppa* gene supported sustained atrial natriuretic peptide hormone production for up to four weeks in mice. The therapeutic benefit of *Nppa* sa-mRNA was further validated in a swine model (246), supporting its potential as a less invasive alternative to direct cardiac injection approaches commonly used in regenerative therapies.

## CONCLUDING REMARKS AND FUTURE DIRECTIONS

Decades of work on alphavirus biology have established the framework for contemporary sa-mRNA platforms, revealing how conserved coding and non-coding elements define replicative competence, host interactions, and tissue tropism. Insights into entry pathways, membrane remodeling, innate immune evasion, and RNA synthesis indicate that alphavirus infection is more complex than a simple receptor-centric process and that distinctions between Old World and New World viruses are less absolute than once appreciated. Dissecting how nonstructural proteins and RNA regulatory elements diverge across species and strains will thus be critical for predicting alphavirus emergence and for rationally engineering sa-mRNA backbones that improve antigen expression while minimizing off-target effects.

Several key questions now define the next phase of sa-mRNA development. First, the fidelity of the error-prone nsP4 RdRp in replicating long heterologous payloads, and its impact on antigen integrity and quasispecies diversity, remain incompletely defined. Chronic CHIKV disease is characterized by macrophage reservoirs of viral RNA that are thought to sustain joint inflammation through persistent CD4^+^ T cell recruitment (247). Although the mechanisms underlying alphavirus persistence are not fully understood, prolonged sa-mRNA activity may similarly generate aberrant DI-like RNA species or mutant payloads capable of redirecting host immune responses toward non-protective outcomes in prophylactic settings. Accordingly, next-generation sa-mRNA platforms should prioritize the development of high-fidelity nsP4 variants or inducible regulatory systems that provide tighter control over gene expression during extended replication cycles.

Second, the relative immunogenicity of the nonstructural replicase proteins versus the encoded antigen, and the extent to which anti-backbone immunity may limit homologous or heterologous boosting, require systematic evaluation. Emerging evidence demonstrating anti-replicase immunity emphasizes the need to expand the repertoire of candidate backbones beyond the commonly used VEEV, SINV, and SFV systems (234). At present, our knowledge of T cell epitopes targeting nonstructural proteins remains limited, although genomic surveillance of circulating CHIKV strains proposes nsP3 as a prominent driver of viral evolution alongside E1 and E2 (248). Exploratory efforts to develop broadly protective alphavirus vaccines have highlighted conserved, immunostimulatory epitopes within nsP2, nsP3, and nsP4 (249). In this context, the flexibility in permitting diverse chimeric P123–nsP4 configurations or taRNA hybrid systems provides a practical strategy to mitigate anti-vector immunity (109, 198).

Third, defining the kinetics and magnitude of replicase activity, innate immune activation, and the counteracting viral antagonism mediated by nsPs and CSEs during sa-mRNA amplification will be critical for optimizing existing backbones and extending the platform to alphaviruses not naturally adapted to humans. A clearer understanding of how and when sa-mRNA-driven antigen expression is turned off will also inform broader questions surrounding the unexpectedly high rates of chronic disease observed following infection with arthritogenic alphaviruses. The observation that many CHIKV replicase molecules do not directly participate in spherule formation or RNA synthesis suggests that the nonstructural proteins may perform additional regulatory or immunomodulatory functions (250).

From a platform engineering perspective, the VEE complex represents a largely underexplored group of alphaviruses that exhibit antigen expression dynamics similar to those of VEEV itself (230). Likewise, next-generation sa-mRNA platforms should more systematically evaluate backbones derived from the SFV complex and related Old World alphaviruses. Future studies will need to reconcile the propensity of arthritogenic alphaviruses to establish chronic infection with the paradoxically strong innate immune restriction often encountered when these viruses are deployed as replicon systems. If appropriately harnessed, Old World alphaviruses may provide favorable platforms for sustained antigen expression in therapeutic settings such as cancer immunotherapy. Conversely, nsP2 variants that only partially inhibit type I IFN signaling may support prolonged, low-level immunogen expression, thereby promoting more durable adaptive immune memory. Given recent findings that ectopically expressed alphavirus replicases can differentially convert host RNA templates into immunostimulatory RNAs (38), it will also be important to determine whether similar processes occur during prophylactic and therapeutic sa-mRNA use. Such template promiscuity may contribute to backbone-specific differences in self-adjuvanticity, with direct implications for immune priming and durability in prophylactic applications.

Finally, more comprehensive investigation of the nsP3 host interactome will be required to better define its roles in innate immune activation, tissue tropism, and RNA persistence. Analysis of RSEs within the 3′ UTR may further clarify mechanisms regulating sa-mRNA stability and turnover *in vivo*. In parallel, the expanding application of alphavirus-derived sa-mRNAs in veterinary vaccinology (251, 252), where dose sparing, rapid reprogrammability, and accelerated regulatory pathways are advantageous, provides a valuable proving ground for next-generation platform designs while underscoring the importance of understanding host range and cell- and tissue-specific tropism.

Together, integration of alphavirus molecular virology with RNA engineering and systems-level host profiling promises to guide the rational evolution of sa-mRNA technologies toward greater efficacy, precision, and clinical versatility.

## References

[1] Strauss JH, Strauss EG. 1994. The alphaviruses: gene expression, replication, and evolution. Microbiol Rev 58:491–562. doi:10.1128/mr.58.3.491-562.1994 7968923 PMC372977

[2] Weaver SC, Winegar R, Manger ID, Forrester NL. 2012. Alphaviruses: population genetics and determinants of emergence. Antiviral Res 94:242–257. doi:10.1016/j.antiviral.2012.04.002 22522323 PMC3737490

[3] Hardy JL, Houk EJ, Kramer LD, Reeves WC. 1983. Intrinsic factors affecting vector competence of mosquitoes for arboviruses. Annu Rev Entomol 28:229–262. doi:10.1146/annurev.en.28.010183.001305 6131642

[4] Forrester NL, Palacios G, Tesh RB, Savji N, Guzman H, Sherman M, Weaver SC, Lipkin WI. 2012. Genome-scale phylogeny of the alphavirus genus suggests a marine origin. J Virol 86:2729–2738. doi:10.1128/JVI.05591-11 22190718 PMC3302268

[5] de Souza WM, Ribeiro GS, de Lima STS, de Jesus R, Moreira FRR, Whittaker C, Sallum MAM, Carrington CVF, Sabino EC, Kitron U, Faria NR, Weaver SC. 2024. Chikungunya: a decade of burden in the Americas. Lancet Reg Health Am 30:100673. doi:10.1016/j.lana.2023.100673 38283942 PMC10820659

[6] Levi LI, Vignuzzi M. 2019. Arthritogenic alphaviruses: a worldwide emerging threat? Microorganisms 7:133. doi:10.3390/microorganisms7050133 31091828 PMC6560413

[7] Weaver SC, Forrester NL. 2015. Chikungunya: evolutionary history and recent epidemic spread. Antiviral Res 120:32–39. doi:10.1016/j.antiviral.2015.04.016 25979669

[8] de Lima Cavalcanti TYV, Pereira MR, de Paula SO, Franca R de O. 2022. A review on chikungunya virus epidemiology, pathogenesis and current vaccine development. Viruses 14:969. doi:10.3390/v14050969 35632709 PMC9147731

[9] Bartholomeeusen K, Daniel M, LaBeaud DA, Gasque P, Peeling RW, Stephenson KE, Ng LFP, Ariën KK. 2023. Chikungunya fever. Nat Rev Dis Primers 9:17. doi:10.1038/s41572-023-00429-2 37024497 PMC11126297

[10] Ribeiro Dos Santos G, Jawed F, Mukandavire C, Deol A, Scarponi D, Mboera LEG, Seruyange E, Poirier MJP, Bosomprah S, Udeze AO, Dellagi K, Hozé N, Chilongola J, Nasrallah GK, Cauchemez S, Salje H. 2025. Global burden of chikungunya virus infections and the potential benefit of vaccination campaigns. Nat Med 31:2342–2349. doi:10.1038/s41591-025-03703-w 40495015 PMC12283390

[11] Mehta R, Gerardin P, de Brito CAA, Soares CN, Ferreira MLB, Solomon T. 2018. The neurological complications of chikungunya virus: a systematic review. Rev Med Virol 28:e1978. doi:10.1002/rmv.1978 29671914 PMC5969245

[12] Elliott KC, Saunders D, Mattapallil JJ. 2026. Venezuelan equine encephalitis virus: novel live-attenuated vaccines for inducing complete protective immunity. Npj Viruses 4:20. doi:10.1038/s44298-026-00186-5 41862693 PMC13004899

[13] Frolov I, Frolova EI. 2022. Molecular virology of chikungunya virus. Curr Top Microbiol Immunol 435:1–31. doi:10.1007/82_2018_146 30599050

[14] Jose J, Snyder JE, Kuhn RJ. 2009. A structural and functional perspective of alphavirus replication and assembly. Future Microbiol 4:837–856. doi:10.2217/fmb.09.59 19722838 PMC2762864

[15] Glasgow GM, McGee MM, Tarbatt CJ, Mooney DA, Sheahan BJ, Atkins GJ. 1998. The Semliki Forest virus vector induces p53-independent apoptosis. J Gen Virol 79:2405–2410. doi:10.1099/0022-1317-79-10-2405 9780045

[16] Shirako Y, Strauss JH. 1994. Regulation of Sindbis virus RNA replication: uncleaved P123 and nsP4 function in minus-strand RNA synthesis, whereas cleaved products from P123 are required for efficient plus-strand RNA synthesis. J Virol 68:1874–1885. doi:10.1128/JVI.68.3.1874-1885.1994 8107248 PMC236650

[17] Lemm JA, Rice CM. 1993. Assembly of functional Sindbis virus RNA replication complexes: requirement for coexpression of P123 and P34. J Virol 67:1905–1915. doi:10.1128/JVI.67.4.1905-1915.1993 8445716 PMC240258

[18] Lemm JA, Rice CM. 1993. Roles of nonstructural polyproteins and cleavage products in regulating Sindbis virus RNA replication and transcription. J Virol 67:1916–1926. doi:10.1128/JVI.67.4.1916-1926.1993 8445717 PMC240259

[19] Melancon P, Garoff H. 1987. Processing of the Semliki Forest virus structural polyprotein: role of the capsid protease. J Virol 61:1301–1309. doi:10.1128/JVI.61.5.1301-1309.1987 3553612 PMC254103

[20] Garoff H, Huylebroeck D, Robinson A, Tillman U, Liljeström P. 1990. The signal sequence of the p62 protein of Semliki Forest virus is involved in initiation but not in completing chain translocation. J Cell Biol 111:867–876. doi:10.1083/jcb.111.3.867 2391367 PMC2116283

[21] Pletnev SV, Zhang W, Mukhopadhyay S, Fisher BR, Hernandez R, Brown DT, Baker TS, Rossmann MG, Kuhn RJ. 2001. Locations of carbohydrate sites on alphavirus glycoproteins show that E1 forms an icosahedral scaffold. Cell 105:127–136. doi:10.1016/s0092-8674(01)00302-6 11301008 PMC4140091

[22] Uchime O, Fields W, Kielian M. 2013. The role of E3 in pH protection during alphavirus assembly and exit. J Virol 87:10255–10262. doi:10.1128/JVI.01507-13 23864626 PMC3754015

[23] Zhang X, Fugère M, Day R, Kielian M. 2003. Furin processing and proteolytic activation of Semliki Forest virus. J Virol 77:2981–2989. doi:10.1128/jvi.77.5.2981-2989.2003 12584323 PMC149766

[24] Firth AE, Chung BY, Fleeton MN, Atkins JF. 2008. Discovery of frameshifting in alphavirus 6K resolves a 20-year enigma. Virol J 5:108. doi:10.1186/1743-422X-5-108 18822126 PMC2569925

[25] Snyder JE, Kulcsar KA, Schultz KLW, Riley CP, Neary JT, Marr S, Jose J, Griffin DE, Kuhn RJ. 2013. Functional characterization of the alphavirus TF protein. J Virol 87:8511–8523. doi:10.1128/JVI.00449-13 23720714 PMC3719798

[26] Rogers KJ, Jones-Burrage S, Maury W, Mukhopadhyay S. 2020. TF protein of Sindbis virus antagonizes host type I interferon responses in a palmitoylation-dependent manner. Virology (Auckl) 542:63–70. doi:10.1016/j.virol.2020.01.001 PMC7024065 32056669

[27] Mukhopadhyay S, Chipman PR, Hong EM, Kuhn RJ, Rossmann MG. 2002. In vitro-assembled alphavirus core-like particles maintain a structure similar to that of nucleocapsid cores in mature virus. J Virol 76:11128–11132. doi:10.1128/JVI.76.21.11128-11132.2002 12368355 PMC136650

[28] Paredes AM, Simon MN, Brown DT. 1992. The mass of the Sindbis virus nucleocapsid suggests it has T = 4 icosahedral symmetry. Virology (Auckl) 187:329–332. doi:10.1016/0042-6822(92)90322-G 1736536

[29] Lescar J, Roussel A, Wien MW, Navaza J, Fuller SD, Wengler G, Wengler G, Rey FA. 2001. The fusion glycoprotein shell of Semliki Forest virus: an icosahedral assembly primed for fusogenic activation at endosomal pH. Cell 105:137–148. doi:10.1016/s0092-8674(01)00303-8 11301009

[30] Mukhopadhyay S, Zhang W, Gabler S, Chipman PR, Strauss EG, Strauss JH, Baker TS, Kuhn RJ, Rossmann MG. 2006. Mapping the structure and function of the E1 and E2 glycoproteins in alphaviruses. Structure 14:63–73. doi:10.1016/j.str.2005.07.025 16407066 PMC2757649

[31] Ahola T, Lampio A, Auvinen P, Kääriäinen L. 1999. Semliki Forest virus mRNA capping enzyme requires association with anionic membrane phospholipids for activity. EMBO J 18:3164–3172. doi:10.1093/emboj/18.11.3164 10357827 PMC1171397

[32] Frolova EI, Gorchakov R, Pereboeva L, Atasheva S, Frolov I. 2010. Functional Sindbis virus replicative complexes are formed at the plasma membrane. J Virol 84:11679–11695. doi:10.1128/JVI.01441-10 20826696 PMC2977861

[33] Bakhache W, Neyret A, Bernard E, Merits A, Briant L. 2020. Palmitoylated cysteines in chikungunya virus nsP1 are critical for targeting to cholesterol-rich plasma membrane microdomains with functional consequences for viral genome replication. J Virol 94:e02183-19. doi:10.1128/JVI.02183-19 32132240 PMC7199415

[34] Jones R, Bragagnolo G, Arranz R, Reguera J. 2021. Capping pores of alphavirus nsP1 gate membranous viral replication factories. Nature 589:615–619. doi:10.1038/s41586-020-3036-8 33328629 PMC7739802

[35] Akhrymuk I, Frolov I, Frolova EI. 2016. Both RIG-I and MDA5 detect alphavirus replication in concentration-dependent mode. Virology (Auckl) 487:230–241. doi:10.1016/j.virol.2015.09.023 PMC4721224 26550947

[36] Pichlmair A, Schulz O, Tan C-P, Rehwinkel J, Kato H, Takeuchi O, Akira S, Way M, Schiavo G, Reis e Sousa C. 2009. Activation of MDA5 requires higher-order RNA structures generated during virus infection. J Virol 83:10761–10769. doi:10.1128/JVI.00770-09 19656871 PMC2753146

[37] Akhrymuk I, Lukash T, Frolov I, Frolova EI. 2019. Novel mutations in nsP2 abolish chikungunya virus-induced transcriptional shutoff and make the virus less cytopathic without affecting its replication rates. J Virol 93:e02062-18. doi:10.1128/JVI.02062-18 30487275 PMC6364006

[38] Omler A, Rutmane A, Mahalingam S, Merits A. 2026. The ability of alphavirus replicases to synthesize non-viral type I interferon-inducing RNAs correlates with viral RNA synthesis and has a diverse impact on virus replication and pathogenicity. J Virol 100:e0216225. doi:10.1128/jvi.02162-25 41603610 PMC12911905

[39] Nikonov A, Mölder T, Sikut R, Kiiver K, Männik A, Toots U, Lulla A, Lulla V, Utt A, Merits A, Ustav M. 2013. RIG-I and MDA-5 detection of viral RNA-dependent RNA polymerase activity restricts positive-strand RNA virus replication. PLoS Pathog 9:e1003610. doi:10.1371/journal.ppat.1003610 24039580 PMC3764220

[40] Webb LG, Veloz J, Pintado-Silva J, Zhu T, Rangel MV, Mutetwa T, Zhang L, Bernal-Rubio D, Figueroa D, Carrau L, Fenutria R, Potla U, Reid SP, Yount JS, Stapleford KA, Aguirre S, Fernandez-Sesma A. 2020. Chikungunya virus antagonizes cGAS-STING mediated type-I interferon responses by degrading cGAS. PLoS Pathog 16:e1008999. doi:10.1371/journal.ppat.1008999 33057424 PMC7591055

[41] Zhang K, Law MCY, Nguyen TM, Tan YB, Wirawan M, Law YS, Jeong LS, Luo D. 2022. Molecular basis of specific viral RNA recognition and 5’-end capping by the chikungunya virus nsP1. Cell Rep 40:111133. doi:10.1016/j.celrep.2022.111133 35905713

[42] Ahola T, Kääriäinen L. 1995. Reaction in alphavirus mRNA capping: formation of a covalent complex of nonstructural protein nsP1 with 7-methyl-GMP. Proc Natl Acad Sci USA 92:507–511. doi:10.1073/pnas.92.2.507 7831320 PMC42770

[43] Wang HL, O’Rear J, Stollar V. 1996. Mutagenesis of the Sindbis virus nsP1 protein: effects on methyltransferase activity and viral infectivity. Virology (Auckl) 217:527–531. doi:10.1006/viro.1996.0147 8610444

[44] Vasiljeva L, Merits A, Auvinen P, Kääriäinen L. 2000. Identification of a novel function of the alphavirus capping apparatus. RNA 5’-triphosphatase activity of Nsp2. J Biol Chem 275:17281–17287. doi:10.1074/jbc.M910340199 10748213

[45] Sokoloski KJ, Haist KC, Morrison TE, Mukhopadhyay S, Hardy RW. 2015. Noncapped alphavirus genomic RNAs and their role during infection. J Virol 89:6080–6092. doi:10.1128/JVI.00553-15 25833042 PMC4442418

[46] LaPointe AT, Moreno-Contreras J, Sokoloski KJ. 2018. Increasing the capping efficiency of the Sindbis virus nsP1 protein negatively affects viral infection. mBio 9:e02342-18. doi:10.1128/mBio.02342-18 30538185 PMC6299483

[47] Karki D, LaPointe AT, Isom C, Thomas M, Sokoloski KJ. 2025. Mechanistic insights into Sindbis virus infection: noncapped genomic RNAs enhance the translation of capped genomic RNAs to promote viral infectivity. Nucleic Acids Res 53:gkae1230. doi:10.1093/nar/gkae1230 39660624 PMC11724270

[48] Law MCY, Zhang K, Tan YB, Nguyen TM, Luo D. 2023. Chikungunya virus nonstructural protein 1 is a versatile RNA capping and decapping enzyme. J Biol Chem 299:105415. doi:10.1016/j.jbc.2023.105415 37918803 PMC10687048

[49] Jones R, Hons M, Rabah N, Zamarreño N, Arranz R, Reguera J. 2023. Structural basis and dynamics of chikungunya alphavirus RNA capping by nsP1 capping pores. Proc Natl Acad Sci USA 120:e2213934120. doi:10.1073/pnas.2213934120 36913573 PMC10041110

[50] Schuberth-Wagner C, Ludwig J, Bruder AK, Herzner A-M, Zillinger T, Goldeck M, Schmidt T, Schmid-Burgk JL, Kerber R, Wolter S, Stümpel J-P, Roth A, Bartok E, Drosten C, Coch C, Hornung V, Barchet W, Kümmerer BM, Hartmann G, Schlee M. 2015. A conserved histidine in the RNA sensor RIG-I controls immune tolerance to N_1_-2'O-methylated self RNA. Immunity 43:41–51. doi:10.1016/j.immuni.2015.06.015 26187414 PMC7128463

[51] Diamond MS. 2014. IFIT1: a dual sensor and effector molecule that detects non-2’-O methylated viral RNA and inhibits its translation. Cytokine Growth Factor Rev 25:543–550. doi:10.1016/j.cytogfr.2014.05.002 24909568 PMC4234691

[52] Hyde JL, Gardner CL, Kimura T, White JP, Liu G, Trobaugh DW, Huang C, Tonelli M, Paessler S, Takeda K, Klimstra WB, Amarasinghe GK, Diamond MS. 2014. A viral RNA structural element alters host recognition of nonself RNA. Science 343:783–787. doi:10.1126/science.1248465 24482115 PMC4209899

[53] Reynaud JM, Kim DY, Atasheva S, Rasalouskaya A, White JP, Diamond MS, Weaver SC, Frolova EI, Frolov I. 2015. IFIT1 differentially interferes with translation and replication of alphavirus genomes and promotes induction of type I interferon. PLoS Pathog 11:e1004863. doi:10.1371/journal.ppat.1004863 25927359 PMC4415776

[54] de Groot RJ, Hardy WR, Shirako Y, Strauss JH. 1990. Cleavage-site preferences of Sindbis virus polyproteins containing the non-structural proteinase. Evidence for temporal regulation of polyprotein processing in vivo. EMBO J 9:2631–2638. doi:10.1002/j.1460-2075.1990.tb07445.x 2142454 PMC552296

[55] Vasiljeva L, Merits A, Golubtsov A, Sizemskaja V, Kääriäinen L, Ahola T. 2003. Regulation of the sequential processing of Semliki Forest virus replicase polyprotein. J Biol Chem 278:41636–41645. doi:10.1074/jbc.M307481200 12917405

[56] Das PK, Merits A, Lulla A. 2014. Functional cross-talk between distant domains of chikungunya virus non-structural protein 2 is decisive for its RNA-modulating activity. J Biol Chem 289:5635–5653. doi:10.1074/jbc.M113.503433 24407286 PMC3937639

[57] Law YS, Wang S, Tan YB, Shih O, Utt A, Goh WY, Lian BJ, Chen MW, Jeng US, Merits A, Luo D. 2021. Interdomain flexibility of chikungunya virus nsP2 helicase-protease differentially influences viral RNA replication and infectivity. J Virol 95:e01470-20. doi:10.1128/JVI.01470-20 33328310 PMC8094934

[58] Law YS, Utt A, Tan YB, Zheng J, Wang S, Chen MW, Griffin PR, Merits A, Luo D. 2019. Structural insights into RNA recognition by the chikungunya virus nsP2 helicase. Proc Natl Acad Sci USA 116:9558–9567. doi:10.1073/pnas.1900656116 31000599 PMC6511008

[59] Shin G, Yost SA, Miller MT, Elrod EJ, Grakoui A, Marcotrigiano J. 2012. Structural and functional insights into alphavirus polyprotein processing and pathogenesis. Proc Natl Acad Sci USA 109:16534–16539. doi:10.1073/pnas.1210418109 23010928 PMC3478664

[60] Hellström K, Kallio K, Utt A, Quirin T, Jokitalo E, Merits A, Ahola T. 2017. Partially uncleaved alphavirus replicase forms spherule structures in the presence and absence of RNA template. J Virol 91:e00787-17. doi:10.1128/JVI.00787-17 28701392 PMC5571266

[61] Garmashova N, Gorchakov R, Frolova E, Frolov I. 2006. Sindbis virus nonstructural protein nsP2 is cytotoxic and inhibits cellular transcription. J Virol 80:5686–5696. doi:10.1128/JVI.02739-05 16731907 PMC1472573

[62] Frolova EI, Fayzulin RZ, Cook SH, Griffin DE, Rice CM, Frolov I. 2002. Roles of nonstructural protein nsP2 and Alpha/Beta interferons in determining the outcome of Sindbis virus infection. J Virol 76:11254–11264. doi:10.1128/jvi.76.22.11254-11264.2002 12388685 PMC136776

[63] Gorchakov R, Frolova E, Sawicki S, Atasheva S, Sawicki D, Frolov I. 2008. A new role for ns polyprotein cleavage in Sindbis virus replication. J Virol 82:6218–6231. doi:10.1128/JVI.02624-07 18417571 PMC2447110

[64] Akhrymuk I, Kulemzin SV, Frolova EI. 2012. Evasion of the innate immune response: the Old World alphavirus nsP2 protein induces rapid degradation of Rpb1, a catalytic subunit of RNA polymerase II. J Virol 86:7180–7191. doi:10.1128/JVI.00541-12 22514352 PMC3416352

[65] Atasheva S, Fish A, Fornerod M, Frolova EI. 2010. Venezuelan equine encephalitis virus capsid protein forms a tetrameric complex with CRM1 and importin α/β that obstructs nuclear pore complex function. J Virol 84:4158–4171. doi:10.1128/JVI.02554-09 20147401 PMC2863722

[66] Abraham R, Hauer D, McPherson RL, Utt A, Kirby IT, Cohen MS, Merits A, Leung AKL, Griffin DE. 2018. ADP-ribosyl-binding and hydrolase activities of the alphavirus nsP3 macrodomain are critical for initiation of virus replication. Proc Natl Acad Sci USA 115:E10457–E10466. doi:10.1073/pnas.1812130115 30322911 PMC6217424

[67] Alhammad YMO, Fehr AR. 2020. The viral macrodomain counters host antiviral ADP-ribosylation. Viruses 12:384. doi:10.3390/v12040384 32244383 PMC7232374

[68] Krieg S, Pott F, Potthoff L, Verheirstraeten M, Bütepage M, Golzmann A, Lippok B, Goffinet C, Lüscher B, Korn P. 2023. Mono-ADP-ribosylation by PARP10 inhibits chikungunya virus nsP2 proteolytic activity and viral replication. Cell Mol Life Sci 80:72. doi:10.1007/s00018-023-04717-8 36840772 PMC9959937

[69] Abraham R, McPherson RL, Dasovich M, Badiee M, Leung AKL, Griffin DE. 2020. Both ADP-ribosyl-binding and hydrolase activities of the alphavirus nsP3 macrodomain affect neurovirulence in mice. mBio 11:e03253-19. doi:10.1128/mBio.03253-19 32047134 PMC7018654

[70] Ware BC, Parks MG, da Silva MOL, Morrison TE. 2024. Chikungunya virus infection disrupts MHC-I antigen presentation via nonstructural protein 2. PLoS Pathog 20:e1011794. doi:10.1371/journal.ppat.1011794 38483968 PMC10965081

[71] Götte B, Liu L, McInerney GM. 2018. The enigmatic alphavirus non-structural protein 3 (nsP3) revealing its secrets at last. Viruses 10:105. doi:10.3390/v10030105 29495654 PMC5869498

[72] Gorchakov R, Garmashova N, Frolova E, Frolov I. 2008. Different types of nsP3-containing protein complexes in Sindbis virus-infected cells. J Virol 82:10088–10101. doi:10.1128/JVI.01011-08 18684830 PMC2566286

[73] Foy NJ, Akhrymuk M, Shustov AV, Frolova EI, Frolov I. 2013. Hypervariable domain of nonstructural protein nsP3 of Venezuelan equine encephalitis virus determines cell-specific mode of virus replication. J Virol 87:7569–7584. doi:10.1128/JVI.00720-13 23637407 PMC3700263

[73] Foy NJ, Akhrymuk M, Akhrymuk I, Atasheva S, Bopda-Waffo A, Frolov I, Frolova EI. 2013. Hypervariable domains of nsP3 proteins of New World and Old World alphaviruses mediate formation of distinct, virus-specific protein complexes. J Virol 87:1997–2010. doi:10.1128/JVI.02853-12 23221551 PMC3571466

[75] Byers NM, Burns PL, Stuchlik O, Reed MS, Ledermann JP, Pohl J, Powers AM. 2023. Identification of mosquito proteins that differentially interact with alphavirus nonstructural protein 3, a determinant of vector specificity. PLoS Negl Trop Dis 17:e0011028. doi:10.1371/journal.pntd.0011028 36696390 PMC9876241

[76] Strauss EG, Levinson R, Rice CM, Dalrymple J, Strauss JH. 1988. Nonstructural proteins nsP3 and nsP4 of Ross River and O’Nyong-nyong viruses: sequence and comparison with those of other alphaviruses. Virology (Auckl) 164:265–274. doi:10.1016/0042-6822(88)90644-7 2834873

[77] Saxton-Shaw KD, Ledermann JP, Borland EM, Stovall JL, Mossel EC, Singh AJ, Wilusz J, Powers AM. 2013. O’nyong nyong virus molecular determinants of unique vector specificity reside in non-structural protein 3. PLoS Negl Trop Dis 7:e1931. doi:10.1371/journal.pntd.0001931 23359824 PMC3554527

[78] Kim DY, Reynaud JM, Rasalouskaya A, Akhrymuk I, Mobley JA, Frolov I, Frolova EI. 2016. New World and Old World alphaviruses have evolved to exploit different components of stress granules, FXR and G3BP proteins, for assembly of viral replication complexes. PLoS Pathog 12:e1005810. doi:10.1371/journal.ppat.1005810 27509095 PMC4980055

[79] Scholte FEM, Tas A, Albulescu IC, Žusinaite E, Merits A, Snijder EJ, van Hemert MJ. 2015. Stress granule components G3BP1 and G3BP2 play a proviral role early in chikungunya virus replication. J Virol 89:4457–4469. doi:10.1128/JVI.03612-14 25653451 PMC4442398

[80] Panas MD, Ahola T, McInerney GM. 2014. The C-terminal repeat domains of nsP3 from the Old World alphaviruses bind directly to G3BP. J Virol 88:5888–5893. doi:10.1128/JVI.00439-14 24623412 PMC4019107

[81] Fros JJ, Domeradzka NE, Baggen J, Geertsema C, Flipse J, Vlak JM, Pijlman GP. 2012. Chikungunya virus nsP3 blocks stress granule assembly by recruitment of G3BP into cytoplasmic foci. J Virol 86:10873–10879. doi:10.1128/JVI.01506-12 22837213 PMC3457282

[82] Götte B, Utt A, Fragkoudis R, Merits A, McInerney GM. 2020. Sensitivity of alphaviruses to G3BP deletion correlates with efficiency of replicase polyprotein processing. J Virol 94:e01681-19. doi:10.1128/JVI.01681-19 31941782 PMC7081891

[83] Frolova EI, Palchevska O, Dominguez F, Frolov I. 2023. Alphavirus-induced transcriptional and translational shutoffs play major roles in blocking the formation of stress granules. J Virol 97:e0097923. doi:10.1128/jvi.00979-23 37902397 PMC10688339

[84] Neuvonen M, Kazlauskas A, Martikainen M, Hinkkanen A, Ahola T, Saksela K. 2011. SH3 domain-mediated recruitment of host cell amphiphysins by alphavirus nsP3 promotes viral RNA replication. PLoS Pathog 7:e1002383. doi:10.1371/journal.ppat.1002383 22114558 PMC3219718

[85] Mutso M, Morro AM, Smedberg C, Kasvandik S, Aquilimeba M, Teppor M, Tarve L, Lulla A, Lulla V, Saul S, Thaa B, McInerney GM, Merits A, Varjak M. 2018. Mutation of CD2AP and SH3KBP1 binding motif in alphavirus nsP3 hypervariable domain results in attenuated virus. Viruses 10:226. doi:10.3390/v10050226 29702546 PMC5977219

[86] Matkovic R, Bernard E, Fontanel S, Eldin P, Chazal N, Hassan Hersi D, Merits A, Péloponèse J-M Jr, Briant L. 2019. The host DHX9 DExH-box helicase is recruited to chikungunya virus replication complexes for optimal genomic RNA translation. J Virol 93:e01764-18. doi:10.1128/JVI.01764-18 30463980 PMC6364007

[87] Lukash T, Agback T, Dominguez F, Shiliaev N, Meshram C, Frolova EI, Agback P, Frolov I. 2020. Structural and functional characterization of host FHL1 protein interaction with hypervariable domain of chikungunya virus nsP3 protein. J Virol 95:e01672-20. doi:10.1128/JVI.01672-20 33055253 PMC7737738

[88] Meertens L, Hafirassou ML, Couderc T, Bonnet-Madin L, Kril V, Kümmerer BM, Labeau A, Brugier A, Simon-Loriere E, Burlaud-Gaillard J, et al.. 2019. FHL1 is a major host factor for chikungunya virus infection. Nature 574:259–263. doi:10.1038/s41586-019-1578-4 31554973

[89] Ng WH, Liu X, Ling ZL, Santos CNO, Magalhães LS, Kueh AJ, Herold MJ, Taylor A, Freitas JR, Koit S, Wang S, Lloyd AR, Teixeira MM, Merits A, Almeida RP, King NJC, Mahalingam S. 2023. FHL1 promotes chikungunya and o’nyong-nyong virus infection and pathogenesis with implications for alphavirus vaccine design. Nat Commun 14:6605. doi:10.1038/s41467-023-42330-2 37884534 PMC10603155

[90] Sun C, Gardner CL, Watson AM, Ryman KD, Klimstra WB. 2014. Stable, high-level expression of reporter proteins from improved alphavirus expression vectors to track replication and dissemination during encephalitic and arthritogenic disease. J Virol 88:2035–2046. doi:10.1128/JVI.02990-13 24307590 PMC3911548

[91] Tamberg N, Lulla V, Fragkoudis R, Lulla A, Fazakerley JK, Merits A. 2007. Insertion of EGFP into the replicase gene of Semliki Forest virus results in a novel, genetically stable marker virus. J Gen Virol 88:1225–1230. doi:10.1099/vir.0.82436-0 17374766 PMC2274952

[92] Hallengärd D, Kakoulidou M, Lulla A, Kümmerer BM, Johansson DX, Mutso M, Lulla V, Fazakerley JK, Roques P, Le Grand R, Merits A, Liljeström P. 2014. Novel attenuated chikungunya vaccine candidates elicit protective immunity in C57BL/6 mice. J Virol 88:2858–2866. doi:10.1128/JVI.03453-13 24371047 PMC3958085

[93] Mazzon M, Castro C, Thaa B, Liu L, Mutso M, Liu X, Mahalingam S, Griffin JL, Marsh M, McInerney GM. 2018. Alphavirus-induced hyperactivation of PI3K/AKT directs pro-viral metabolic changes. PLoS Pathog 14:e1006835. doi:10.1371/journal.ppat.1006835 29377936 PMC5805360

[94] Kril V, Hons M, Amadori C, Zimberger C, Couture L, Bouery Y, Burlaud-Gaillard J, Karpov A, Ptchelkine D, Thienel AL, Kümmerer BM, Desfosses A, Jones R, Roingeard P, Meertens L, Amara A, Reguera J. 2024. Alphavirus nsP3 organizes into tubular scaffolds essential for infection and the cytoplasmic granule architecture. Nat Commun 15:8106. doi:10.1038/s41467-024-51952-z 39285216 PMC11405681

[95] Luo D, Tan YB, Law MCY, Jin J. 2025. A structural perspective on the alphavirus life cycle. Annu Rev Virol 12:299–314. doi:10.1146/annurev-virology-093022-010359 40720808

[96] Gao Y, Goonawardane N, Ward J, Tuplin A, Harris M. 2019. Multiple roles of the non-structural protein 3 (nsP3) alphavirus unique domain (AUD) during chikungunya virus genome replication and transcription. PLoS Pathog 15:e1007239. doi:10.1371/journal.ppat.1007239 30668592 PMC6358111

[97] Bhattacharya T, Freeman TS, Alleman EM, Wang F, Chechik L, Emerman M, Myles KM, Malik HS. 2025. The Sindbis virus nsP3 opal codon protects viral RNA and fitness by maintaining replication spherule integrity. bioRxiv:2025.09.27.679005. doi:10.1101/2025.09.27.679005

[98] Li R, Sun K, Tuplin A, Harris M. 2023. A structural and functional analysis of opal stop codon translational readthrough during chikungunya virus replication. J Gen Virol 104. doi:10.1099/jgv.0.001909 PMC7615711 37862073

[99] Jones JE, Long KM, Whitmore AC, Sanders W, Thurlow LR, Brown JA, Morrison CR, Vincent H, Peck KM, Browning C, Moorman N, Lim JK, Heise MT. 2017. Disruption of the opal stop codon attenuates chikungunya virus-induced arthritis and pathology. mBio 8:e01456-17. doi:10.1128/mBio.01456-17 29138302 PMC5686535

[100] Li GP, Rice CM. 1989. Mutagenesis of the in-frame opal termination codon preceding nsP4 of Sindbis virus: studies of translational readthrough and its effect on virus replication. J Virol 63:1326–1337. doi:10.1128/JVI.63.3.1326-1337.1989 2521676 PMC247830

[101] Bhattacharya T, Alleman EM, Freeman TS, Noyola AC, Emerman M, Malik HS. 2025. A conserved opal termination codon optimizes a temperature-dependent trade-off between protein production and processing in alphaviruses. Sci Adv 11:eads7933. doi:10.1126/sciadv.ads7933 40249804 PMC13105319

[102] Hwang Kim K, Rümenapf T, Strauss EG, Strauss JH. 2004. Regulation of Semliki Forest virus RNA replication: a model for the control of alphavirus pathogenesis in invertebrate hosts. Virology (Auckl) 323:153–163. doi:10.1016/j.virol.2004.03.009 15165827

[103] Bakovic A, Bhalla N, Kortchak S, Sun C, Zhou W, Ahmed A, Risner K, Klimstra WB, Narayanan A. 2020. Venezuelan equine encephalitis virus nsP3 phosphorylation can be mediated by IKKβ kinase activity and abrogation of phosphorylation inhibits negative-strand synthesis. Viruses 12:1021. doi:10.3390/v12091021 32933112 PMC7551587

[104] Amaya M, Voss K, Sampey G, Senina S, de la Fuente C, Mueller C, Calvert V, Kehn-Hall K, Carpenter C, Kashanchi F, Bailey C, Mogelsvang S, Petricoin E, Narayanan A. 2014. The role of IKKβ in Venezuelan equine encephalitis virus infection. PLoS One 9:e86745. doi:10.1371/journal.pone.0086745 24586253 PMC3929299

[105] Rubach JK, Wasik BR, Rupp JC, Kuhn RJ, Hardy RW, Smith JL. 2009. Characterization of purified Sindbis virus nsP4 RNA-dependent RNA polymerase activity in vitro. Virology (Auckl) 384:201–208. doi:10.1016/j.virol.2008.10.030 PMC3107704 19036396

[106] Shirako Y, Strauss JH. 1998. Requirement for an aromatic amino acid or histidine at the N terminus of Sindbis virus RNA polymerase. J Virol 72:2310–2315. doi:10.1128/JVI.72.3.2310-2315.1998 9499091 PMC109530

[107] de Groot RJ, Rümenapf T, Kuhn RJ, Strauss EG, Strauss JH. 1991. Sindbis virus RNA polymerase is degraded by the N-end rule pathway. Proc Natl Acad Sci USA 88:8967–8971. doi:10.1073/pnas.88.20.8967 1924357 PMC52632

[108] Shirako Y, Strauss EG, Strauss JH. 2000. Suppressor mutations that allow sindbis virus RNA polymerase to function with nonaromatic amino acids at the N-terminus: evidence for interaction between nsP1 and nsP4 in minus-strand RNA synthesis. Virology (Auckl) 276:148–160. doi:10.1006/viro.2000.0544 11022003

[109] Lello LS, Bartholomeeusen K, Wang S, Coppens S, Fragkoudis R, Alphey L, Ariën KK, Merits A, Utt A. 2021. nsP4 is a major determinant of alphavirus replicase activity and template selectivity. J Virol 95:e0035521. doi:10.1128/JVI.00355-21 34319783 PMC8475546

[110] Tomar S, Hardy RW, Smith JL, Kuhn RJ. 2006. Catalytic core of alphavirus nonstructural protein nsP4 possesses terminal adenylyltransferase activity. J Virol 80:9962–9969. doi:10.1128/JVI.01067-06 17005674 PMC1617302

[111] Poirier EZ, Mounce BC, Rozen-Gagnon K, Hooikaas PJ, Stapleford KA, Moratorio G, Vignuzzi M. 2016. Low-fidelity polymerases of alphaviruses recombine at higher rates to overproduce defective interfering particles. J Virol 90:2446–2454. doi:10.1128/JVI.02921-15 PMC4810721 26676773

[112] Rozen-Gagnon K, Stapleford KA, Mongelli V, Blanc H, Failloux AB, Saleh MC, Vignuzzi M. 2014. Alphavirus mutator variants present host-specific defects and attenuation in mammalian and insect models. PLoS Pathog 10:e1003877. doi:10.1371/journal.ppat.1003877 24453971 PMC3894214

[113] Coffey LL, Beeharry Y, Bordería AV, Blanc H, Vignuzzi M. 2011. Arbovirus high fidelity variant loses fitness in mosquitoes and mice. Proc Natl Acad Sci USA 108:16038–16043. doi:10.1073/pnas.1111650108 21896755 PMC3179076

[114] Riemersma KK, Steiner C, Singapuri A, Coffey LL. 2019. Chikungunya virus fidelity variants exhibit differential attenuation and population diversity in cell culture and adult mice. J Virol 93:e01606-18. doi:10.1128/JVI.01606-18 30429348 PMC6340026

[115] Yin P, Sobolik EB, May NA, Wang S, Fayed A, Vyshenska D, Drobish AM, Parks MG, Lello LS, Merits A, Morrison TE, Greninger AL, Kielian M. 2025. Mutations in chikungunya virus nsP4 decrease viral fitness and sensitivity to the broad-spectrum antiviral 4′-fluorouridine. PLoS Pathog 21:e1012859. doi:10.1371/journal.ppat.1012859 39804924 PMC11759387

[116] Levis R, Weiss BG, Tsiang M, Huang H, Schlesinger S. 1986. Deletion mapping of Sindbis virus DI RNAs derived from cDNAs defines the sequences essential for replication and packaging. Cell 44:137–145. doi:10.1016/0092-8674(86)90492-7 3753584

[117] Ou JH, Rice CM, Dalgarno L, Strauss EG, Strauss JH. 1982. Sequence studies of several alphavirus genomic RNAs in the region containing the start of the subgenomic RNA. Proc Natl Acad Sci USA 79:5235–5239. doi:10.1073/pnas.79.17.5235 6291034 PMC346870

[118] Levis R, Huang H, Schlesinger S. 1987. Engineered defective interfering RNAs of Sindbis virus express bacterial chloramphenicol acetyltransferase in avian cells. Proc Natl Acad Sci USA 84:4811–4815. doi:10.1073/pnas.84.14.4811 2440030 PMC305195

[119] Rice CM, Levis R, Strauss JH, Huang HV. 1987. Production of infectious RNA transcripts from Sindbis virus cDNA clones: mapping of lethal mutations, rescue of a temperature-sensitive marker, and in vitro mutagenesis to generate defined mutants. J Virol 61:3809–3819. doi:10.1128/JVI.61.12.3809-3819.1987 3479621 PMC255997

[120] Xiong C, Levis R, Shen P, Schlesinger S, Rice CM, Huang HV. 1989. Sindbis virus: an efficient, broad host range vector for gene expression in animal cells. Science 243:1188–1191. doi:10.1126/science.2922607 2922607

[121] Weiss BG, Schlesinger S. 1991. Recombination between Sindbis virus RNAs. J Virol 65:4017–4025. doi:10.1128/JVI.65.8.4017-4025.1991 2072444 PMC248832

[122] Schlesinger S, Dubensky TW. 1999. Alphavirus vectors for gene expression and vaccines. Curr Opin Biotechnol 10:434–439. doi:10.1016/s0958-1669(99)00006-3 10508626

[123] Pushko P, Parker M, Ludwig GV, Davis NL, Johnston RE, Smith JF. 1997. Replicon-helper systems from attenuated Venezuelan equine encephalitis virus: expression of heterologous genes in vitro and immunization against heterologous pathogens in vivo. Virology (Auckl) 239:389–401. doi:10.1006/viro.1997.8878 9434729

[124] Smerdou C, Liljeström P. 1999. Two-helper RNA system for production of recombinant Semliki Forest virus particles. J Virol 73:1092–1098. doi:10.1128/JVI.73.2.1092-1098.1999 9882310 PMC103929

[125] Frolov I, Schlesinger S. 1994. Translation of Sindbis virus mRNA: effects of sequences downstream of the initiating codon. J Virol 68:8111–8117. doi:10.1128/JVI.68.12.8111-8117.1994 7966601 PMC237275

[126] Sjöberg EM, Suomalainen M, Garoff H. 1994. A significantly improved Semliki Forest virus expression system based on translation enhancer segments from the viral capsid gene. Biotechnology (N Y) 12:1127–1131. doi:10.1038/nbt1194-1127 7765556

[127] Dominguez F, Palchevska O, Frolova EI, Frolov I. 2023. Alphavirus-based replicons demonstrate different interactions with host cells and can be optimized to increase protein expression. J Virol 97:e0122523. doi:10.1128/jvi.01225-23 37877718 PMC10688356

[128] Frolova E, Frolov I, Schlesinger S. 1997. Packaging signals in alphaviruses. J Virol 71:248–258. doi:10.1128/JVI.71.1.248-258.1997 8985344 PMC191045

[129] White CL, Thomson M, Dimmock NJ. 1998. Deletion analysis of a defective interfering Semliki Forest virus RNA genome defines a region in the nsP2 sequence that is required for efficient packaging of the genome into virus particles. J Virol 72:4320–4326. doi:10.1128/JVI.72.5.4320-4326.1998 9557722 PMC109662

[130] Bredenbeek PJ, Frolov I, Rice CM, Schlesinger S. 1993. Sindbis virus expression vectors: packaging of RNA replicons by using defective helper RNAs. J Virol 67:6439–6446. doi:10.1128/JVI.67.11.6439-6446.1993 8411346 PMC238079

[131] Kim DY, Atasheva S, Foy NJ, Wang E, Frolova EI, Weaver S, Frolov I. 2011. Design of chimeric alphaviruses with a programmed, attenuated, cell type-restricted phenotype. J Virol 85:4363–4376. doi:10.1128/JVI.00065-11 21345954 PMC3126257

[132] Hahn CS, Hahn YS, Braciale TJ, Rice CM. 1992. Infectious Sindbis virus transient expression vectors for studying antigen processing and presentation. Proc Natl Acad Sci USA 89:2679–2683. doi:10.1073/pnas.89.7.2679 1372987 PMC48725

[133] Jackson AC, SenGupta SK, Smith JF. 1991. Pathogenesis of Venezuelan equine encephalitis virus infection in mice and hamsters. Vet Pathol 28:410–418. doi:10.1177/030098589102800509 1750167

[134] MacDonald GH, Johnston RE. 2000. Role of dendritic cell targeting in Venezuelan equine encephalitis virus pathogenesis. J Virol 74:914–922. doi:10.1128/jvi.74.2.914-922.2000 10623754 PMC111612

[135] Grieder FB, Davis NL, Aronson JF, Charles PC, Sellon DC, Suzuki K, Johnston RE. 1995. Specific restrictions in the progression of Venezuelan equine encephalitis virus-induced disease resulting from single amino acid changes in the glycoproteins. Virology (Auckl) 206:994–1006. doi:10.1006/viro.1995.1022 7856110

[136] Davis NL, Brown KW, Johnston RE. 1996. A viral vaccine vector that expresses foreign genes in lymph nodes and protects against mucosal challenge. J Virol 70:3781–3787. doi:10.1128/jvi.70.6.3781-3787.1996 8648713 PMC190254

[137] Liljeström P. 1994. Alphavirus expression systems. Curr Opin Biotechnol 5:495–500. doi:10.1016/0958-1669(94)90063-9 7765462

[138] Gardner JP, Frolov I, Perri S, Ji Y, MacKichan ML, zur Megede J, Chen M, Belli BA, Driver DA, Sherrill S, Greer CE, Otten GR, Barnett SW, Liu MA, Dubensky TW, Polo JM. 2000. Infection of human dendritic cells by a sindbis virus replicon vector is determined by a single amino acid substitution in the E2 glycoprotein. J Virol 74:11849–11857. doi:10.1128/JVI.74.24.11849-11857.2000 11090185 PMC112468

[139] Perri S, Greer CE, Thudium K, Doe B, Legg H, Liu H, Romero RE, Tang Z, Bin Q, Dubensky TW, Vajdy M, Otten GR, Polo JM. 2003. An alphavirus replicon particle chimera derived from venezuelan equine encephalitis and sindbis viruses is a potent gene-based vaccine delivery vector. J Virol 77:10394–10403. doi:10.1128/jvi.77.19.10394-10403.2003 12970424 PMC228391

[140] Polo JM, Belli BA, Driver DA, Frolov I, Sherrill S, Hariharan MJ, Townsend K, Perri S, Mento SJ, Jolly DJ, Chang SMW, Schlesinger S, Dubensky TW. 1999. Stable alphavirus packaging cell lines for Sindbis virus and Semliki Forest virus-derived vectors. Proc Natl Acad Sci USA 96:4598–4603. doi:10.1073/pnas.96.8.4598 10200308 PMC16378

[141] Chaudhary N, Weissman D, Whitehead KA. 2021. mRNA vaccines for infectious diseases: principles, delivery and clinical translation. Nat Rev Drug Discov 20:817–838. doi:10.1038/s41573-021-00283-5 34433919 PMC8386155

[142] Ssentongo P, Ssentongo AE, Voleti N, Groff D, Sun A, Ba DM, Nunez J, Parent LJ, Chinchilli VM, Paules CI. 2022. SARS-CoV-2 vaccine effectiveness against infection, symptomatic and severe COVID-19: a systematic review and meta-analysis. BMC Infect Dis 22:439. doi:10.1186/s12879-022-07418-y 35525973 PMC9077344

[143] Comes JDG, Pijlman GP, Hick TAH. 2023. Rise of the RNA machines – self-amplification in mRNA vaccine design. Trends Biotechnol 41:1417–1429. doi:10.1016/j.tibtech.2023.05.007 37328401 PMC10266560

[144] Casmil IC, Jin J, Won EJ, Huang C, Liao S, Cha-Molstad H, Blakney AK. 2025. The advent of clinical self-amplifying RNA vaccines. Mol Ther 33:2565–2582. doi:10.1016/j.ymthe.2025.03.060 40186353 PMC12172325

[145] Frolov I, Hoffman TA, Prágai BM, Dryga SA, Huang HV, Schlesinger S, Rice CM. 1996. Alphavirus-based expression vectors: strategies and applications. Proc Natl Acad Sci USA 93:11371–11377. doi:10.1073/pnas.93.21.11371 8876142 PMC38064

[146] Ljungberg K, Liljeström P. 2015. Self-replicating alphavirus RNA vaccines. Expert Rev Vaccines 14:177–194. doi:10.1586/14760584.2015.965690 25269775

[147] Rayner JO, Dryga SA, Kamrud KI. 2002. Alphavirus vectors and vaccination. Rev Med Virol 12:279–296. doi:10.1002/rmv.360 12211042

[148] Chang C, Patel H, Ferrari A, Scalzo T, Petkov D, Xu H, Rossignol E, Palladino G, Wen Y. 2025. sa-mRNA influenza vaccine raises a higher and more durable immune response than mRNA vaccine in preclinical models. Vaccine (Auckl) 51:126883. doi:10.1016/j.vaccine.2025.126883 39956088

[149] Geall AJ, Verma A, Otten GR, Shaw CA, Hekele A, Banerjee K, Cu Y, Beard CW, Brito LA, Krucker T, O’Hagan DT, Singh M, Mason PW, Valiante NM, Dormitzer PR, Barnett SW, Rappuoli R, Ulmer JB, Mandl CW. 2012. Nonviral delivery of self-amplifying RNA vaccines. Proc Natl Acad Sci USA 109:14604–14609. doi:10.1073/pnas.1209367109 22908294 PMC3437863

[150] Vallet T, Vignuzzi M. 2025. Self-amplifying RNA: advantages and challenges of a versatile platform for vaccine development. Viruses 17:566. doi:10.3390/v17040566 40285008 PMC12031284

[151] Machado BAS, Hodel KVS, Fonseca LMDS, Mascarenhas LAB, Andrade LPC da S, Rocha VPC, Soares MBP, Berglund P, Duthie MS, Reed SG, Badaró R. 2021. The importance of RNA-based vaccines in the fight against COVID-19: an overview. Vaccines (Basel) 9:1345. doi:10.3390/vaccines9111345 34835276 PMC8623509

[152] Lundstrom K. 2020. Self-amplifying RNA viruses as RNA vaccines. Int J Mol Sci 21:5130. doi:10.3390/ijms21145130 32698494 PMC7404065

[153] Amano T, Yu H, Amano M, Leyder E, Badiola M, Ray P, Kim J, Ko AC, Achour A, Weng N-P, Kochba E, Levin Y, Ko MSH. 2023. Controllable self-replicating RNA vaccine delivered intradermally elicits predominantly cellular immunity. iScience 26:106335. doi:10.1016/j.isci.2023.106335 36968065 PMC10034440

[154] Berge TO, Banks IS, Tigertt WD. 1961. Attenuation of Venezuelan equine encephalomyelitis virus by in vitro cultivation in guinea-pig heart cells 1. Am J Epidemiol 73:209–218. doi:10.1093/oxfordjournals.aje.a120178

[155] Kulasegaran-Shylini R, Thiviyanathan V, Gorenstein DG, Frolov I. 2009. The 5′UTR-specific mutation in VEEV TC-83 genome has a strong effect on RNA replication and subgenomic RNA synthesis, but not on translation of the encoded proteins. Virology (Auckl) 387:211–221. doi:10.1016/j.virol.2009.02.027 PMC2675632 19278709

[156] Kinney RM, Chang GJ, Tsuchiya KR, Sneider JM, Roehrig JT, Woodward TM, Trent DW. 1993. Attenuation of Venezuelan equine encephalitis virus strain TC-83 is encoded by the 5’-noncoding region and the E2 envelope glycoprotein. J Virol 67:1269–1277. doi:10.1128/jvi.67.3.1269-1277.1993 7679745 PMC237493

[157] Spotts DR, Reich RM, Kalkhan MA, Kinney RM, Roehrig JT. 1998. Resistance to alpha/beta interferons correlates with the epizootic and virulence potential of Venezuelan equine encephalitis viruses and is determined by the 5′ noncoding region and glycoproteins. J Virol 72:10286–10291. doi:10.1128/JVI.72.12.10286-10291.1998 9811777 PMC110615

[158] White LJ, Wang JG, Davis NL, Johnston RE. 2001. Role of alpha/beta interferon in Venezuelan equine encephalitis virus pathogenesis: effect of an attenuating mutation in the 5′ untranslated region. J Virol 75:3706–3718. doi:10.1128/JVI.75.8.3706-3718.2001 11264360 PMC114862

[159] Petrakova O, Volkova E, Gorchakov R, Paessler S, Kinney RM, Frolov I. 2005. Noncytopathic replication of Venezuelan equine encephalitis virus and eastern equine encephalitis virus replicons in Mammalian cells. J Virol 79:7597–7608. doi:10.1128/JVI.79.12.7597-7608.2005 15919912 PMC1143662

[160] Frolov I, Agapov E, Hoffman TA Jr, Prágai BM, Lippa M, Schlesinger S, Rice CM. 1999. Selection of RNA replicons capable of persistent noncytopathic replication in mammalian cells. J Virol 73:3854–3865. doi:10.1128/JVI.73.5.3854-3865.1999 10196280 PMC104163

[161] Perri S, Driver DA, Gardner JP, Sherrill S, Belli BA, Dubensky TW Jr, Polo JM. 2000. Replicon vectors derived from Sindbis virus and Semliki forest virus that establish persistent replication in host cells. J Virol 74:9802–9807. doi:10.1128/jvi.74.20.9802-9807.2000 11000258 PMC112418

[162] Utt A, Das PK, Varjak M, Lulla V, Lulla A, Merits A. 2015. Mutations conferring a noncytotoxic phenotype on chikungunya virus replicons compromise enzymatic properties of nonstructural protein 2. J Virol 89:3145–3162. doi:10.1128/JVI.03213-14 25552719 PMC4337533

[163] Casales E, Rodriguez-Madoz JR, Ruiz-Guillen M, Razquin N, Cuevas Y, Prieto J, Smerdou C. 2008. Development of a new noncytopathic Semliki Forest virus vector providing high expression levels and stability. Virology (Auckl) 376:242–251. doi:10.1016/j.virol.2008.03.016 18442838

[164] Gorchakov R, Frolova E, Williams BRG, Rice CM, Frolov I. 2004. PKR-dependent and -independent mechanisms are involved in translational shutoff during Sindbis virus infection. J Virol 78:8455–8467. doi:10.1128/JVI.78.16.8455-8467.2004 15280454 PMC479073

[165] Rathore APS, Ng M-L, Vasudevan SG. 2013. Differential unfolded protein response during Chikungunya and Sindbis virus infection: CHIKV nsP4 suppresses eIF2α phosphorylation. Virol J 10:36. doi:10.1186/1743-422X-10-36 23356742 PMC3605262

[166] Ventoso I, Sanz MA, Molina S, Berlanga JJ, Carrasco L, Esteban M. 2006. Translational resistance of late alphavirus mRNA to eIF2α phosphorylation: a strategy to overcome the antiviral effect of protein kinase PKR. Genes Dev 20:87–100. doi:10.1101/gad.357006 16391235 PMC1356103

[167] Sanz MA, González Almela E, Carrasco L. 2017. Translation of Sindbis subgenomic mRNA is independent of eIF2, eIF2A and eIF2D. Sci Rep 7:43876. doi:10.1038/srep43876 28240315 PMC5327398

[168] Sanz MA, Castelló A, Ventoso I, Berlanga JJ, Carrasco L. 2009. Dual mechanism for the translation of subgenomic mRNA from Sindbis virus in infected and uninfected cells. PLoS One 4:e4772. doi:10.1371/journal.pone.0004772 19274090 PMC2651626

[169] Garmashova N, Gorchakov R, Volkova E, Paessler S, Frolova E, Frolov I. 2007. The Old World and New World alphaviruses use different virus-specific proteins for induction of transcriptional shutoff. J Virol 81:2472–2484. doi:10.1128/JVI.02073-06 17108023 PMC1865960

[170] Breakwell L, Dosenovic P, Karlsson Hedestam GB, D’Amato M, Liljeström P, Fazakerley J, McInerney GM. 2007. Semliki Forest virus nonstructural protein 2 is involved in suppression of the type I interferon response. J Virol 81:8677–8684. doi:10.1128/JVI.02411-06 17553895 PMC1951358

[171] Fros JJ, Liu WJ, Prow NA, Geertsema C, Ligtenberg M, Vanlandingham DL, Schnettler E, Vlak JM, Suhrbier A, Khromykh AA, Pijlman GP. 2010. Chikungunya virus nonstructural protein 2 inhibits type I/II interferon-stimulated JAK-STAT signaling. J Virol 84:10877–10887. doi:10.1128/JVI.00949-10 20686047 PMC2950581

[172] Göertz GP, McNally KL, Robertson SJ, Best SM, Pijlman GP, Fros JJ. 2018. The methyltransferase-like domain of chikungunya virus nsP2 inhibits the interferon response by promoting the nuclear export of STAT1. J Virol 92:e01008-18. doi:10.1128/JVI.01008-18 29925658 PMC6096799

[173] Fros JJ, van der Maten E, Vlak JM, Pijlman GP. 2013. The C-terminal domain of chikungunya virus nsP2 independently governs viral RNA replication, cytopathicity, and inhibition of interferon signaling. J Virol 87:10394–10400. doi:10.1128/JVI.00884-13 23864632 PMC3753987

[174] Frolov I, Garmashova N, Atasheva S, Frolova EI. 2009. Random insertion mutagenesis of sindbis virus nonstructural protein 2 and selection of variants incapable of downregulating cellular transcription. J Virol 83:9031–9044. doi:10.1128/JVI.00850-09 19570872 PMC2738241

[175] Akhrymuk I, Frolov I, Frolova EI. 2018. Sindbis virus infection causes cell death by nsP2-induced transcriptional shutoff or by nsP3-dependent translational shutoff. J Virol 92:e01388-18. doi:10.1128/JVI.01388-18 30232189 PMC6232463

[176] Treffers EE, Tas A, Scholte FEM, de Ru AH, Snijder EJ, van Veelen PA, van Hemert MJ. 2023. The alphavirus nonstructural protein 2 NTPase induces a host translational shut-off through phosphorylation of eEF2 via cAMP-PKA-eEF2K signaling. PLoS Pathog 19:e1011179. doi:10.1371/journal.ppat.1011179 36848386 PMC9997916

[177] Bhalla N, Sun C, Metthew Lam LK, Gardner CL, Ryman KD, Klimstra WB. 2016. Host translation shutoff mediated by non-structural protein 2 is a critical factor in the antiviral state resistance of Venezuelan equine encephalitis virus. Virology (Auckl) 496:147–165. doi:10.1016/j.virol.2016.06.005 PMC5821108 27318152

[178] Gong Y, Yong D, Liu G, Xu J, Ding J, Jia W. 2024. A novel self-amplifying mRNA with decreased cytotoxicity and enhanced protein expression by macrodomain mutations. Adv Sci (Weinh) 11:e2402936. doi:10.1002/advs.202402936 39313862 PMC11578319

[179] Kulasegaran-Shylini R, Atasheva S, Gorenstein DG, Frolov I. 2009. Structural and functional elements of the promoter encoded by the 5’ untranslated region of the Venezuelan equine encephalitis virus genome. J Virol 83:8327–8339. doi:10.1128/JVI.00586-09 19515761 PMC2738147

[180] Ou JH, Strauss EG, Strauss JH. 1983. The 5’-terminal sequences of the genomic RNAs of several alphaviruses. J Mol Biol 168:1–15. doi:10.1016/s0022-2836(83)80319-2 6308269

[181] Kim DY, Atasheva S, McAuley AJ, Plante JA, Frolova EI, Beasley DWC, Frolov I. 2014. Enhancement of protein expression by alphavirus replicons by designing self-replicating subgenomic RNAs. Proc Natl Acad Sci USA 111:10708–10713. doi:10.1073/pnas.1408677111 25002490 PMC4115546

[182] Pfeffer M, Kinney RM, Kaaden OR. 1998. The alphavirus 3′-nontranslated region: size heterogeneity and arrangement of repeated sequence elements. Virology (Auckl) 240:100–108. doi:10.1006/viro.1997.8907 9448694

[183] Hyde JL, Chen R, Trobaugh DW, Diamond MS, Weaver SC, Klimstra WB, Wilusz J. 2015. The 5′ and 3′ ends of alphavirus RNAs – Non-coding is not non-functional. Virus Res 206:99–107. doi:10.1016/j.virusres.2015.01.016 25630058 PMC4654126

[184] Chen R, Wang E, Tsetsarkin KA, Weaver SC. 2013. Chikungunya virus 3′ untranslated region: adaptation to mosquitoes and a population bottleneck as major evolutionary forces. PLoS Pathog 9:e1003591. doi:10.1371/journal.ppat.1003591 24009512 PMC3757053

[185] Garneau NL, Sokoloski KJ, Opyrchal M, Neff CP, Wilusz CJ, Wilusz J. 2008. The 3’ untranslated region of sindbis virus represses deadenylation of viral transcripts in mosquito and mammalian cells. J Virol 82:880–892. doi:10.1128/JVI.01205-07 17977976 PMC2224598

[186] Sokoloski KJ, Dickson AM, Chaskey EL, Garneau NL, Wilusz CJ, Wilusz J. 2010. Sindbis virus usurps the cellular HuR protein to stabilize its transcripts and promote productive infections in mammalian and mosquito cells. Cell Host Microbe 8:196–207. doi:10.1016/j.chom.2010.07.003 20709296 PMC2929003

[187] Morley VJ, Noval MG, Chen R, Weaver SC, Vignuzzi M, Stapleford KA, Turner PE. 2018. Chikungunya virus evolution following a large 3’UTR deletion results in host-specific molecular changes in protein-coding regions. Virus Evol 4:vey012. doi:10.1093/ve/vey012 29942653 PMC6007266

[188] Filomatori CV, Merwaiss F, Bardossy ES, Alvarez DE. 2021. Impact of alphavirus 3’UTR plasticity on mosquito transmission. Semin Cell Dev Biol 111:148–155. doi:10.1016/j.semcdb.2020.07.006 32665176

[189] Bardossy ES, Volpe S, Alvarez DE, Filomatori CV. 2023. A conserved Y-shaped RNA structure in the 3’UTR of chikungunya virus genome as a host-specialized element that modulates viral replication and evolution. PLoS Pathog 19:e1011352. doi:10.1371/journal.ppat.1011352 37126493 PMC10174580

[190] Garcia-Moreno M, Sanz MA, Carrasco L. 2016. A viral mRNA motif at the 3'-untranslated region that confers translatability in a cell-specific manner. Implications for virus evolution. Sci Rep 6:19217. doi:10.1038/srep19217 26755446 PMC4709744

[191] Hardy RW, Rice CM. 2005. Requirements at the 3’ end of the sindbis virus genome for efficient synthesis of minus-strand RNA. J Virol 79:4630–4639. doi:10.1128/JVI.79.8.4630-4639.2005 15795249 PMC1069581

[192] Hickson SE, Brekke E, Schwerk J, Saluhke I, Zaver S, Woodward J, Savan R, Hyde JL. 2025. Sequence diversity in the 3' untranslated region of alphavirus modulates IFIT2-dependent restriction in a cell type-dependent manner. J Interferon Cytokine Res 45:133–149. doi:10.1089/jir.2024.0229 40079162 PMC12491954

[193] McGee JE, Kirsch JR, Kenney D, Cerbo F, Chavez EC, Shih T-Y, Douam F, Wong WW, Grinstaff MW. 2025. Complete substitution with modified nucleotides in self-amplifying RNA suppresses the interferon response and increases potency. Nat Biotechnol 43:720–726. doi:10.1038/s41587-024-02306-z 38977924 PMC11707045

[194] Eaton BT, Faulkner P. 1972. Heterogeneity in the poly(A) content of the genome of sindbis virus. Virology (Auckl) 50:865–873. doi:10.1016/0042-6822(72)90440-0 4640690

[195] Kääriäinen L, Takkinen K, Keränen S, Söderlund H. 1987. Replication of the genome of alphaviruses. J Cell Sci 1987:231–250. doi:10.1242/jcs.1987.Supplement_7.17 2846593

[196] Yıldız A, Răileanu C, Beissert T. 2024. Trans-amplifying RNA: a journey from alphavirus research to future vaccines. Viruses 16:503. doi:10.3390/v16040503 38675846 PMC11055088

[197] Perkovic M, Gawletta S, Hempel T, Brill S, Nett E, Sahin U, Beissert T. 2023. A trans-amplifying RNA simplified to essential elements is highly replicative and robustly immunogenic in mice. Mol Ther 31:1636–1646. doi:10.1016/j.ymthe.2023.01.019 36694464 PMC10277886

[198] Lello LS, Utt A, Bartholomeeusen K, Wang S, Rausalu K, Kendall C, Coppens S, Fragkoudis R, Tuplin A, Alphey L, Ariën KK, Merits A. 2020. Cross-utilisation of template RNAs by alphavirus replicases. PLoS Pathog 16:e1008825. doi:10.1371/journal.ppat.1008825 32886709 PMC7498090

[199] Gorchakov R, Hardy R, Rice CM, Frolov I. 2004. Selection of functional 5’ cis-acting elements promoting efficient sindbis virus genome replication. J Virol 78:61–75. doi:10.1128/jvi.78.1.61-75.2004 14671088 PMC303405

[200] Hassett KJ, Rajlic IL, Bahl K, White R, Cowens K, Jacquinet E, Burke KE. 2024. mRNA vaccine trafficking and resulting protein expression after intramuscular administration. Mol Ther Nucleic Acids 35:102083. doi:10.1016/j.omtn.2023.102083 38161733 PMC10755037

[201] Hou X, Zaks T, Langer R, Dong Y. 2021. Lipid nanoparticles for mRNA delivery. Nat Rev Mater 6:1078–1094. doi:10.1038/s41578-021-00358-0 34394960 PMC8353930

[202] Di J, Du Z, Wu K, Jin S, Wang X, Li T, Xu Y. 2022. Biodistribution and non-linear gene expression of mRNA LNPs affected by delivery route and particle size. Pharm Res 39:105–114. doi:10.1007/s11095-022-03166-5 35080707 PMC8791091

[203] Vasileva O, Zaborova O, Shmykov B, Ivanov R, Reshetnikov V. 2024. Composition of lipid nanoparticles for targeted delivery: application to mRNA therapeutics. Front Pharmacol 15:1466337. doi:10.3389/fphar.2024.1466337 39508050 PMC11537937

[204] Pateev I, Seregina K, Ivanov R, Reshetnikov V. 2023. Biodistribution of RNA vaccines and of their products: evidence from human and animal studies. Biomedicines 12:59. doi:10.3390/biomedicines12010059 38255166 PMC10812935

[205] Cui X, Vervaeke P, Gao Y, Opsomer L, Sun Q, Snoeck J, Devriendt B, Zhong Z, Sanders NN. 2024. Immunogenicity and biodistribution of lipid nanoparticle formulated self-amplifying mRNA vaccines against H5 avian influenza. NPJ Vaccines 9:138. doi:10.1038/s41541-024-00932-x 39097672 PMC11298010

[206] Maruggi G, Mallett CP, Westerbeck JW, Chen T, Lofano G, Friedrich K, Qu L, Sun JT, McAuliffe J, Kanitkar A, et al.. 2022. A self-amplifying mRNA SARS-CoV-2 vaccine candidate induces safe and robust protective immunity in preclinical models. Mol Ther 30:1897–1912. doi:10.1016/j.ymthe.2022.01.001 34990810 PMC8721936

[207] Ruiz-Guillen M, Gabev E, Quetglas JI, Casales E, Ballesteros-Briones MC, Poutou J, Aranda A, Martisova E, Bezunartea J, Ondiviela M, Prieto J, Hernandez-Alcoceba R, Abrescia NGA, Smerdou C. 2016. Capsid-deficient alphaviruses generate propagative infectious microvesicles at the plasma membrane. Cell Mol Life Sci 73:3897–3916. doi:10.1007/s00018-016-2230-1 27117550 PMC7079800

[208] Ruiz-Guillen M, Abrescia NGA, Smerdou C. 2017. Neurotropic alphaviruses can propagate without capsid. Oncotarget 8:8999–9000. doi:10.18632/oncotarget.13993 28129638 PMC5354702

[209] Zhang YN, Deng CL, Li JQ, Li N, Zhang QY, Ye HQ, Yuan ZM, Zhang B. 2019. Infectious Chikungunya virus (CHIKV) with a complete capsid deletion: a new approach for a CHIKV vaccine. J Virol 93:e00504-19. doi:10.1128/JVI.00504-19 31092567 PMC6639289

[210] Zhang HQ, Zhang YN, Deng CL, Zhu QX, Zhang ZR, Li XD, Yuan ZM, Zhang B. 2024. Rational design of self-amplifying virus-like vesicles with Ebola virus glycoprotein as vaccines. Mol Ther 32:3695–3711. doi:10.1016/j.ymthe.2024.08.026 39217415 PMC11489537

[211] Aïqui-Reboul-Paviet O, Bakhache W, Bernard E, Holsteyn L, Neyret A, Briant L. 2024. The Rac1-PAK1-Arp2/3 signaling axis regulates CHIKV nsP1-induced filopodia and optimal viral genome replication. J Virol 98:e0061224. doi:10.1128/jvi.00612-24 39297643 PMC11495065

[212] Laakkonen P, Auvinen P, Kujala P, Kääriäinen L. 1998. Alphavirus replicase protein NSP1 induces filopodia and rearrangement of actin filaments. J Virol 72:10265–10269. doi:10.1128/JVI.72.12.10265-10269.1998 9811773 PMC110611

[213] Martin CK, Wan JJ, Yin P, Morrison TE, Messer WB, Rivera-Amill V, Lai JR, Grau N, Rey FA, Couderc T, Lecuit M, Kielian M. 2025. The alphavirus determinants of intercellular long extension formation. mBio 16:e0198624. doi:10.1128/mbio.01986-24 39699169 PMC11796390

[214] Yin P, Davenport BJ, Wan JJ, Kim AS, Diamond MS, Ware BC, Tong K, Couderc T, Lecuit M, Lai JR, Morrison TE, Kielian M. 2023. Chikungunya virus cell-to-cell transmission is mediated by intercellular extensions in vitro and in vivo. Nat Microbiol 8:1653–1667. doi:10.1038/s41564-023-01449-0 37591996 PMC10956380

[215] Lampio A, Kilpeläinen I, Pesonen S, Karhi K, Auvinen P, Somerharju P, Kääriäinen L. 2000. Membrane binding mechanism of an RNA virus-capping enzyme. J Biol Chem 275:37853–37859. doi:10.1074/jbc.M004865200 10984480

[216] Spuul P, Salonen A, Merits A, Jokitalo E, Kääriäinen L, Ahola T. 2007. Role of the amphipathic peptide of Semliki Forest virus replicase protein nsP1 in membrane association and virus replication. J Virol 81:872–883. doi:10.1128/JVI.01785-06 17093195 PMC1797454

[217] Charles PC, Walters E, Margolis F, Johnston RE. 1995. Mechanism of neuroinvasion of Venezuelan equine encephalitis virus in the mouse. Virology (Auckl) 208:662–671. doi:10.1006/viro.1995.1197 7747437

[218] Schäfer A, Brooke CB, Whitmore AC, Johnston RE. 2011. The role of the blood-brain barrier during Venezuelan equine encephalitis virus infection. J Virol 85:10682–10690. doi:10.1128/JVI.05032-11 21849461 PMC3187510

[219] Ferguson MC, Saul S, Fragkoudis R, Weisheit S, Cox J, Patabendige A, Sherwood K, Watson M, Merits A, Fazakerley JK. 2015. Ability of the encephalitic arbovirus semliki forest virus to cross the blood-brain barrier is determined by the charge of the E2 glycoprotein. J Virol 89:7536–7549. doi:10.1128/JVI.03645-14 25972559 PMC4505677

[220] Weger-Lucarelli J, Aliota MT, Wlodarchak N, Kamlangdee A, Swanson R, Osorio JE. 2015. Dissecting the role of E2 protein domains in alphavirus pathogenicity. J Virol 90:2418–2433. doi:10.1128/JVI.02792-15 26676771 PMC4810718

[221] Salimi H, Cain MD, Jiang X, Roth RA, Beatty WL, Sun C, Klimstra WB, Hou J, Klein RS. 2020. Encephalitic alphaviruses exploit caveola-mediated transcytosis at the blood-brain barrier for central nervous system entry. mBio 11:e02731-19. doi:10.1128/mBio.02731-19 32047126 PMC7018649

[222] Alvarez PA, Tang A, Winters DM, Kaushal P, Medina A, Nieto MV, Kaczor-Urbanowicz KE, St Amant F, Reyes BR, Kaake RM, Fregoso OI, Pyle AD, Bouhaddou M, Tang H, Li MMH. 2025. Old World alphaviruses use distinct mechanisms to infect brain microvascular endothelial cells for neuroinvasion. Cell Rep 44:116305. doi:10.1016/j.celrep.2025.116305 40987287 PMC12617940

[223] Hick TAH, Geertsema C, Nguyen W, Bishop CR, van Oosten L, Abbo SR, Dumenil T, van Kuppeveld FJM, Langereis MA, Rawle DJ, Tang B, Yan K, van Oers MM, Suhrbier A, Pijlman GP. 2024. Safety concern of recombination between self-amplifying mRNA vaccines and viruses is mitigated in vivo. Mol Ther 32:2519–2534. doi:10.1016/j.ymthe.2024.06.019 38894543 PMC11405153

[224] Hick T.A.H, Geertsema C, Nijland R, Pijlman GP. 2024. Packaging of alphavirus-based self-amplifying mRNA yields replication-competent virus through a mechanism of aberrant homologous RNA recombination. mBio 15:e0249424. doi:10.1128/mbio.02494-24 39320113 PMC11481888

[225] Reitmayer CM, Levitt E, Basu S, Atkinson B, Fragkoudis R, Merits A, Lumley S, Larner W, Diaz AV, Rooney S, Thomas CJE, von Wyschetzki K, Rausalu K, Alphey L. 2023. Mimicking superinfection exclusion disrupts alphavirus infection and transmission in the yellow fever mosquito Aedes aegypti. Proc Natl Acad Sci USA 120:e2303080120. doi:10.1073/pnas.2303080120 37669371 PMC10500260

[226] Ehrengruber MU, Goldin AL. 2007. Semliki Forest virus vectors with mutations in the nonstructural protein 2 gene permit extended superinfection of neuronal and non-neuronal cells. J Neurovirol 13:353–363. doi:10.1080/13550280701393204 17849319

[227] Hick TAH, Zotler T, Bosveld D, Geertsema C, van Oers MM, Pijlman GP. 2024. Venezuelan equine encephalitis virus non-structural protein 3 dictates superinfection exclusion in mammalian cells. Npj Viruses 2:43. doi:10.1038/s44298-024-00055-z 40295792 PMC11721081

[228] Yousefpour P, Gregory JR, Si K, Lonzaric J, Li Y, Wang J, Qureshi K, Ledbetter A, Lemnios AA, Dye J, Remba TK, Yeung R, Güereca M, Rodriguez L, Zhang Y, Wu S, Dong Y, Weiss R, Irvine DJ. 2025. Engineering gene expression dynamics via self-amplifying RNA with drug-responsive non-structural proteins. bioRxiv:2025.06.08.658495. doi:10.1101/2025.06.08.658495

[229] Bell CL, Yu D, Smolke CD, Geall AJ, Beard CW, Mason PW. 2015. Control of alphavirus-based gene expression using engineered riboswitches. Virology (Auckl) 483:302–311. doi:10.1016/j.virol.2015.04.023 26005949

[230] Sun Z, Liu Y, Zhang H, Ge T, Pan Y, Liu Y, Wu M, Shan T, Zhu G, Wu Q, Chen K. 2026. A novel alphavirus-based saRNA platform for enhanced vaccination efficacy and organ-specific targeting. Vaccine 72:128055. doi:10.1016/j.vaccine.2025.128055 41349243

[231] Ylösmäki E, Martikainen M, Hinkkanen A, Saksela K. 2013. Attenuation of Semliki Forest virus neurovirulence by microRNA-mediated detargeting. J Virol 87:335–344. doi:10.1128/JVI.01940-12 23077310 PMC3536368

[232] Kamrud KI, Coffield VM, Owens G, Goodman C, Alterson K, Custer M, Murphy MA, Lewis W, Timberlake S, Wansley EK, Berglund P, Smith J. 2010. In vitro and in vivo characterization of microRNA-targeted alphavirus replicon and helper RNAs. J Virol 84:7713–7725. doi:10.1128/JVI.00310-10 20504925 PMC2897628

[233] Szurgot I, Ljungberg K, Kümmerer BM, Liljeström P. 2020. Infectious RNA vaccine protects mice against chikungunya virus infection. Sci Rep 10:21076. doi:10.1038/s41598-020-78009-7 33273501 PMC7712826

[234] Cui X, Amelinck L, Gillon O, Catani JPP, Gao Y, Sun Q, De Sutter E, Vervaeke P, Snoeck J, Lienenklaus S, Saelens X, Zhong Z, Sanders NN. 2025. Impact of pre-existing anti-replicase immunity on the efficacy of self-amplifying mRNA vaccines. Nat Commun 17:278. doi:10.1038/s41467-025-66993-1 41353442 PMC12783641

[235] Casmil IC, Bathula NV, Huang C, Wayne CJ, Cairns ES, Friesen JJ, Soriano SK, Liao S, Ho CH, Kong KYS, Blakney AK. 2025. Alphaviral backbone of self-amplifying RNA enhances protein expression and immunogenicity against SARS-CoV-2 antigen. Mol Ther 33:514–528. doi:10.1016/j.ymthe.2024.12.055 39741413 PMC11852984

[236] Agarwal R, Ha C, Côrtes FH, Lee Y, Martínez-Pérez A, Gálvez RI, Castillo IN, Phillips EJ, Mallal SA, Balmaseda A, Harris E, Romero-Vivas CM, Premkumar L, Falconar AK, Grifoni A, Sette A, Weiskopf D. 2025. Identification of immunogenic and cross-reactive chikungunya virus epitopes for CD4^+^ T cells in chronic chikungunya disease. Nat Commun 16:5756. doi:10.1038/s41467-025-60862-7 40592820 PMC12218217

[237] Ramos da Silva J, Bitencourt Rodrigues K, Formoso Pelegrin G, Silva Sales N, Muramatsu H, de Oliveira Silva M, Porchia BFMM, Moreno ACR, Aps LRMM, Venceslau-Carvalho AA, Tombácz I, Fotoran WL, Karikó K, Lin PJC, Tam YK, de Oliveira Diniz M, Pardi N, de Souza Ferreira LC. 2023. Single immunizations of self-amplifying or non-replicating mRNA-LNP vaccines control HPV-associated tumors in mice. Sci Transl Med 15:eabn3464. doi:10.1126/scitranslmed.abn3464 36867683

[238] Aunins EA, Phan AT, Alameh M-G, Dwivedi G, Cruz-Morales E, Christian DA, Tam Y, Bunkofske ME, Peñafiel AZ, O’Dea KM, Merolle M, Furey C, Scott P, Vonderheide RH, Hensley SE, Kedl RM, Weissman D, Hunter CA. 2025. An Il12 mRNA-LNP adjuvant enhances mRNA vaccine–induced CD8 T cell responses. Sci Immunol 10:eads1328. doi:10.1126/sciimmunol.ads1328 40478935 PMC13012528

[239] Peng K, Zhao X, Li H, Fu YX, Liang Y. 2025. Membrane-IL12 adjuvant mRNA vaccine polarizes pre-effector T cells for optimized tumor control. J Exp Med 222:e20241454. doi:10.1084/jem.20241454 40479650 PMC12143231

[240] Miyake-Stoner SJ, Maine CJ, Chou AC, Dailey GP, Spasova DS, Domingo CC, Sparks J, Picarda G, Lyerly HK, Crosby EJ, Geall AJ, Wang NS, Hartman ZC, Aliahmad P. 2026. Enhanced efficacy of a next-generation EEEV self-replicating RNA platform for combination cancer immunotherapies. Mol Ther:S1525-0016(26)00212-1. doi:10.1016/j.ymthe.2026.03.026 PMC13102400 41889175

[241] Quetglas JI, Ruiz-Guillen M, Aranda A, Casales E, Bezunartea J, Smerdou C. 2010. Alphavirus vectors for cancer therapy. Virus Res 153:179–196. doi:10.1016/j.virusres.2010.07.027 20692305

[242] Morse MA, Crosby EJ, Force J, Osada T, Hobeika AC, Hartman ZC, Berglund P, Smith J, Lyerly HK. 2023. Clinical trials of self-replicating RNA-based cancer vaccines. Cancer Gene Ther 30:803–811. doi:10.1038/s41417-023-00587-1 36765179 PMC9911953

[243] Xia Y, Su M, Ye Z, Du F, Wang X, Guan D, Zhang X, Rao Z, Ning P. 2025. An epigenetic regulator synergizes with alphavirus-mediated gene therapy via biomimetic delivery for enhanced cancer therapy. Trends Biotechnol 43:1196–1214. doi:10.1016/j.tibtech.2025.01.007 39955233

[244] Vanluchene H, Gillon O, Peynshaert K, De Smedt SC, Sanders N, Raemdonck K, Remaut K. 2024. Less is more: self-amplifying mRNA becomes self-killing upon dose escalation in immune-competent retinal cells. Eur J Pharm Biopharm 196:114204. doi:10.1016/j.ejpb.2024.114204 38302048

[245] Sanchez-David RY, Le HD, Nemegeer J, Gonçalves A, Merits A, Maillard PV. 2025. Nodamuravirus protein B2 boosts self-amplifying mRNA efficacy by overcoming innate immune barriers. bioRxiv. doi:10.1101/2025.06.27.661928

[246] Zhang K, Tao H, Zhu D, Yue Z, Hu S, Wu Y, Yan N, Hu Y, Liu S, Liu M, Vahl TP, Ranard LS, Cheng X, Romanov A, Liu J, Zhang SW, Li Y, Lu C, Shen M, Lewis A, Huang K, Cheng K. 2026. Single intramuscular injection of self-amplifying RNA of Nppa to treat myocardial infarction. Science 391:edau9394. doi:10.1126/science.adu9394 41785353 PMC13124201

[247] Zarrella KM, Sheridan RM, Ware BC, Davenport BJ, da Silva MOL, Vyshenska D, Martin AU, May NA, Fish ER, Weiskopf D, Hesselberth JR, Streblow DN, Greninger AL, Morrison TE. 2026. Chikungunya virus persists in joint-associated macrophages and promotes chronic disease in mice. Nat Microbiol 11:1302–1317. doi:10.1038/s41564-026-02303-9 41922840 PMC13171603

[248] Rodríguez-Aguilar ED, Gutiérrez-Millán E, Rodríguez MH. 2024. Accurate recapitulation of chikungunya virus complete coding sequence phylogeny using variable genome regions for genomic surveillance. Viruses 16:926. doi:10.3390/v16060926 38932218 PMC11209212

[249] Versiani AF, McCaffrey P, Ribeiro-Filho HV, Silva NIO, Lopes-de-Oliveira PS, Carrera JP, Nogueira ML, Marques RE, Rossi SL, Vasilakis N. 2026. Integrated reiterative pipeline for rapid epitope-based pan-alphavirus vaccines. Sci Adv 12:eaeb2066. doi:10.1126/sciadv.aeb2066 41811958 PMC12978219

[250] Tan YB, Chmielewski D, Law MCY, Zhang K, He Y, Chen M, Jin J, Luo D. 2022. Molecular architecture of the chikungunya virus replication complex. Sci Adv 8:eadd2536. doi:10.1126/sciadv.add2536 36449616 PMC9710867

[251] Ling T, Xin Z, Huan-Huan L, Ya-Ting Z, Ya-Mei L, Rong-Li G, Damiani AM, Sai-Sai C, Chuan-Jian Z, Rui D, Shu-Yu M, Jia-Li Y, Qian-Qian Z, Ruo-Nan T, Osterrieder N, Shu-Hua X, Ji-Chun W. 2025. A self-amplifying mRNA vaccine expressing PRV gD induces robust immunity against virulent mutants. NPJ Vaccines 10:193. doi:10.1038/s41541-025-01251-5 40813770 PMC12354905

[252] Li H-H, Tong L, Chen S-S, Bi Z-X, Hu G, Chen W-L, Yu J-L, Tao R-N, Huang T, Xia S-H, Osterrieder N, Wang J-C. 2026. A self-amplifying mRNA vaccine for infectious laryngotracheitis virus (ILTV) induces efficient protective immunity. Poult Sci 105:106632. doi:10.1016/j.psj.2026.106632 41713095 PMC12926564

